# Comparative Analyses of the Transcriptome and Proteome of *Escherichia coli* C321.△A and Further Improving Its Noncanonical Amino Acids Containing Protein Expression Ability by Integration of T7 RNA Polymerase

**DOI:** 10.3389/fmicb.2021.744284

**Published:** 2021-09-29

**Authors:** Huawei Yi, Jing Zhang, Famin Ke, Xiurong Guo, Jian Yang, Peijuan Xie, Li Liu, Qin Wang, Xiaowei Gao

**Affiliations:** ^1^ Clinical Laboratory, The First Affiliated Hospital of Yangtze University, Jingzhou, China; ^2^ School of Pharmacy, Southwest Medical University, Luzhou, China; ^3^ Department of Chemistry, Zhejiang University, Hangzhou, China

**Keywords:** genetic code expansion, noncanonical amino acids, transcriptome, proteome, *Escherichia coli*, T7 RNA polymerase

## Abstract

Incorporation of noncanonical amino acids (ncAAs) into proteins has been proven to be a powerful tool to manipulate protein structure and function, and to investigate many biological processes. Improving the yields of ncAA-containing proteins is of great significance in industrial-scale applications. *Escherichia coli* C321.ΔA was generated by the replacement of all known amber codons and the deletion of RF1 in the genome and has been proven to be an ideal host for ncAA-containing protein expression using genetic code expansion. In this study, we investigated the transcriptome and proteome profiles of this first codon reassignment strain and found that some functions and metabolic pathways were differentially expressed when compared with those of its parent strain. Genes involved in carbohydrate and energy metabolism were remarkably downregulated. Our results may provide important clues about the growth defects in *E. coli* C321.ΔA. Furthermore, we improved the yields of ncAA-containing proteins in *E. coli* C321.ΔA by integrating the T7 RNA polymerase system.

## Introduction

DNA and RNA are genetic information carriers that determine the lengths and sequences of proteins (Crick, 1970). As the main gene products, proteins participate in nearly every cellular process and have a wide range of functions, including signal transduction, transcriptional regulation, catalytic reaction, and cytoskeleton formation. Despite the diverse functional roles of proteins, nearly all of them are composed of 20 canonical amino acid building blocks (Polycarpo et al., 2004). In nature, posttranslational modifications of proteins, such as methylation, phosphorylation, and glycosylation at specific residues, usually occur. These modifications can augment new chemistries in proteins and make the functions of proteins more versatile (Seo and Lee, 2004). It has also been proven that that site-specific incorporation of noncanonical amino acids (ncAAs) with different functional structures into proteins has the potential to enhance the physical, chemical, and biological properties of proteins, and even generate new functions (Chin, 2014; Xiao and Schultz, 2016).

Genetic code expansion has been proven to be a powerful tool to site-specifically incorporate ncAAs into proteins *in vivo*. More than 200 ncAAs with different functional groups have been genetically encoded in both prokaryotic and eukaryotic organisms with high fidelity and efficiency (Xiao and Schultz, 2016). A heterogeneous and orthogonal tRNA/synthetase pair is needed to insert ncAAs of interest in response to a nonsense or frameshift codon. Although genetic code expansion has been successfully applied in yeast, mammalian cells, and even animals, *Escherichia coli* remains an attractive expression host because of its robustness, low cost, facile manipulation, and high levels of protein expression (Chin, 2014; Wals and Ovaa, 2014). Several orthogonal tRNA/synthetase pairs from *Methanocaldococcus jannaschii*, *Methanosarcina barkeri*, *Methanosarcina mazei*, *Saccharomyces cerevisiae*, and *Pyrococcus horikoshii* have been successfully engineered in *E. coli* to genetically encode a large number of ncAAs with novel functions, including unique chemical reactivities, metal binding, photo-crosslinking, photocaging, and fluorescence (Liu and Schultz, 2010).

To date, the amber codon UAG is the most frequently used codon for ncAA incorporation in genetic code expansion. Naturally, UAG is recognized by release factor 1 (RF1) and mediates peptide chain synthesis termination during translation in *E. coli* (Ryden et al., 1986). Due to the competition of RF1, the recognition and binding efficiency of UAG by the engineered orthogonal UAG-decoding tRNA-synthetase pair is not sufficient, and the suppression rate for a single amber codon is limited to 10–20% (Young et al., 2010). To address this issue, considerable effort has been made with different strategies, including increasing the copy number of orthogonal synthetase/tRNA genes, modification of Ef-tu (elongation factor thermo-unstable) to accommodate ncAAs-tRNA substrates, engineering of orthogonal ribosomes, and deletion of RF1 in *E. coli* (Wang et al., 2007; Young et al., 2010; Johnson et al., 2011; Park et al., 2011; Chatterjee et al., 2013).

Previously, it was reported that RF1 is essential for *E. coli* to decode UAG and terminate translation, and knockout of the *prfA* gene, encoding RF1, from the *E. coli* genome is lethal (Ryden and Isaksson, 1984). However, Mukai et al. (2010) found that eliminating RF1 could be achieved after engineering seven essential genes: *coaD*, *hda*, *hemA*, *mreC*, *murF*, *lolA*, and *lpxK*, of *E. coli* to end with ochre codon UAA. Johnson et al. (2011) found that RF1 could also be removed by deactivation of the in-frame UAG autoregulation element and mutation of Thr246 to alanine in RF2. Both studies demonstrated that the yields of the ncAA-containing proteins were increased, and the efficiency of incorporation of ncAAs at multiple sites in a single polypeptide was also improved when these RF1 deleted *E. coli* strains were used as expression hosts. However, the growth rates of these *E. coli* strains decreased remarkably.

Deletion of RF1 in *E. coli* would result in C-terminal extensions of many proteins unexpectedly due to the read-through of amber stop codons, which might cause cellular growth defects (Johnson et al., 2011). By using multiplex automated genome engineering and conjugative assembly genome engineering, Lajoie et al. (2013) replaced all known amber codons UAG in *E. coli* MG1655 with synonymous ochre codons UAA and deleted RF1 to generate the strain *E. coli* C321.ΔA. In *E. coli* C321.ΔA, UAG is no longer a stop codon, but a blank codon that can be used to assign unambiguously to a natural amino acid or ncAA. The yields of full-length proteins bearing one or multiple ncAAs were remarkably increased and comparable to those of wild-type proteins when using *E. coli* C321.ΔA as an expression host. Up to 30 ncAAs can be incorporated into a single polypeptide with high yields (~50mg/L) and accuracy (>95%; Amiram et al., 2015). Thus, *E. coli* C321.ΔA is an ideal expression host for genetic code expansion and an ideal chassis cell to reassign other synonymous codon to generate more blank codons.

To *de novo* design and synthesis of non-canonical polymers in living cells, chassis cell with robust ncAAs containing protein expression ability is needed to perform further genomic, metabolic, and protein engineering. It would be helpful to obtain more information about the physiological and biochemical characteristics and reveal the potential effects of codon reassignment on *E. coli*. Considered that codon reassignment in the whole genome may affect gene transcription and translation, we comparatively analyzed the transcriptome and proteome of the first codon reassignment strain *E. coli* C321.ΔA in this study. Because T7 RNA polymerase elongates peptide chains approximately five times faster than the *E. coli* RNA polymerase, the T7 RNA polymerase system has become one of the most widely used expression systems in *E. coli* (Balzer et al., 2013). The pET-derived plasmids based on the T7 RNA polymerase system have become the preferred expression systems in industry due to their high expression capabilities. To be able to use the T7 RNA polymerase system in *E. coli* C321.ΔA, we integrated the T7 RNA polymerase gene generated from *E. coli* BL21(DE3) into the genome of *E. coli* C321.ΔA.exp. We comparatively tested the ability of this strain to express ncAA-containing proteins with pET-derived plasmids and found that the yields of the target proteins were remarkably increased. Thus, the strains *E. coli* C321.ΔA exp T7 obtained in this study could facilitate and further improve the yields of ncAA-containing proteins when used as expression hosts with pET-derived plasmids.

## Materials and Methods

### Materials

FastDigest restriction enzymes were purchased from Fermentas (Burlington, Canada). The EasyGeno Assembly Cloning kit was purchased from Tiangen Biotech (Beijing, China). The 2× Taq PCR Master Mix was purchased from Beijing Solarbio Science & Technology Co., Ltd. (Beijing, China). KOD-Plus-Neo DNA polymerase was purchased from Toyobo Co., Ltd. (Osaka, Japan). Oligonucleotide primers and genes were synthesized by GENEWIZ Bio Inc. (Suzhou, China). All other chemicals and reagents used were of analytical grade.

### Bacteria Strains and Growth Conditions

Detailed information on the strains used in this study is presented in Table 1. *Escherichia coli* EcNR2, *E. coli* C321.ΔA, and *E. coli* C321.ΔA.exp were obtained from Addgene (Watertown, MA, United States). *Escherichia coli* DH10B and *E. coli* BL21(DE3) were purchased from Thermo Fisher Scientific (Rockford, IL, United States) and Novagen (Darmstadt, Germany), respectively. Unless otherwise indicated, bacteria were grown in LB medium at 37 or 30°C (for *E. coli* EcNR2 and *E. coli* C321.ΔA only) supplemented with kanamycin (30μg/ml), spectinomycin (50μg/ml), chloramphenicol (34μg/ml), and/or ampicillin (50μg/ml) when needed.

**Table 1:** Bacterial strains and plasmids used in this study.

Strain or plasmid	Relevant characteristics	Sources
Strains		
*E. coli* EcNR2	*E. coli* MG1655 Δ*mutS*::*cat*Δ(*ybhB-bioAB*)::[λcI857 N(*cro-ea59*)::*tetR*-*bla*]	Addgene (ID: 26931)
*E. coli* C321.ΔA	*E. coli* MG1655Δ (*ybhB-bioAB*)::[λcI857 N(*croea59*)::*tetR-bla*] Δ*prfA* Δ*mutS*::*zeoR*; all 321 TAG codons changed to TAA	Addgene (ID: 48998)
*E. coli* C321.ΔA.exp	*E. coli* MG1655 Δ(*ybhB-bioAB*)::*zeoR* Δ*prfA*; all 321 TAG codons changed to TAA	Addgene (ID: 49018)
*E. coli C321.*ΔA. expT7	*E. coli* MG1655Δ(*ybhB-bioAB*)::*zeoR* Δ*prfA*Δ*lacZ*::*T7*gene1; all 321 TAG codons changed to TAA	This study
*E. coli* DH10B	F^−^ *mcrA* Δ(*mrr-hsdRMS-mcrBC*) φ80*lacZ*ΔM15 Δ*lacX74 recA1 endA1 araD139* Δ (*ara-leu*)7697 *galU galK* λ^−^ *rpsL*(Str^R^) *nupG*	ThermoFisher
*E. coli* BL21(DE3)	*F^−^ ompT hsdS (rB^−^ mB^−^)* gal(λ *cI*857 *ind*1 *Sam*7 *nin*5 *lac* UV5-T7gene1) dcm^+^ *λ(DE3)*	Novagen
Plasmids		
pCAS	Plasmid for CRISPR/Cas9 editing in *E. coli*	Addgene (ID:62225)
pTargetF	Plasmid for CRISPR/Cas9 editing in *E. coli*	Addgene (ID:62226)
pTarget-*lacZ*	pTarget harboring corresponding DNA sequence of *lacZ* sgRNA	This study
pULTRA-CNF	pULTRA plasmid harboring a polyspecific *M. jannaschii* tyrosyl-synthetase/tRNA pair	Addgene (ID: 48215)
pEVOL-pBpF	pEVOL plasmid harboring a tRNA-synthetase pair specific for p-Benzoyl-L-Phenylalanine	Addgene (ID: 31190)
pEVOL-ActK	pEVOL plasmid harboring a tRNA-synthetase pair specific for Nε-acetyl-L-lysine	This study
pULTRA-5HTP	pULTRA plasmid harboring a tRNA-synthetase pair specific for 5-hydroxytryptophan	This study
pBAD24-*GFP*	pBAD24 plasmid harboring the GFP gene	Our lab
pET26b-*GFP*	pET26b plasmid harboring the GFP gene	Our lab
pBAD24-*GFP Y151TAG*	pBAD24 plasmid harboring GFP gene with an amber mutation at site Tyr-151	This study
pET26b-*GFP Y151TAG*	pET26b plasmid harboring GFP gene with an amber mutation at site Tyr-151	This study
pBAD24-*GFP Y39TAG/Y151TAG/Y182TAG*	pBAD24 plasmid harboring GFP gene with amber mutations at site Tyr-39, Tyr-151, Tyr-182	This study
pET26b-*GFP Y39TAG/Y151TAG/Y182TAG*	pET26b plasmid harboring GFP gene with amber mutations at site Tyr-39, Tyr-151, Tyr-182	This study

### Transcriptomic Analyses of *E. coli Ec*NR2 and *E. coli* C321.ΔA

After the OD_600_ values of the culture reached 0.6~0.8, the cells were harvested by centrifugation and washed three times with sterile phosphate buffer at 4°C. Total RNA was extracted from the pellet cells using the RNeasy Plant Mini Kit (Qiagen, CA, United States) according to the manufacturer’s protocol. The purity and concentration of the resulting RNA were determined using a NanoDrop instrument (Thermo Scientific, Wilmington, DE, United States), and the integrity was analyzed using 1% agarose gel electrophoresis. RNA integrity number values were calculated using the Agilent Bioanalyzer 2,100 system (Agilent Technologies, Santa Clara, United States). Ribosomal RNA (rRNA) was depleted from the samples using the Illumina Ribo-Zero rRNA removal kit, and the resulting mRNA was fragmented with an average insert length of approximately 200bp. After construction of the cDNA library using the TruSeq Stranded Total RNA Library Prep Kit (Illumina, San Diego, CA, United States), the sequences were determined using the Illumina HiSeq platform at Majorbio Bio-Pharm Technology Co., Ltd., Shanghai, China. To gain statistical confidence, RNA-seq experiments were performed in three independent biological replicates. The datasets generated for this study can be found in the NCBI with an accession number of PRJNA748036.

The quality of the raw reads was filtered and controlled using SeqPrep^1^ and Sickle software (Version 1.33). The resulting valid sequences were then mapped and aligned to the reference genome (*E. coli* C321.ΔA)^2^ using BLAST^+^ (version 2.9.0) and Bowtie2 (version 2.3.5).^3^ The rRNA contamination assessment was performed by randomly selecting 10,000 raw reads in each sample and aligning them to Rfam database^4^ with blast method. Based on the annotation results, percentage of rRNA in each sample is calculated, which is estimated as rRNA contamination. The bioinformatics analyses were performed using the free online platform of Majorbio Cloud Platform^5^ from Shanghai Majorbio Bio-pharm Technology Co., Ltd. The gene expression level analyses were performed using RSEM tool.^6^ FPKM and TPM method are used to calculated expression level, FPKM represents fragments per kilobase of exon model per million mapped reads, and TPM represents transcript per million mapped reads. The FPKM and TPM are able to eliminate the influence of different gene length and sequencing discrepancy on the calculation of gene expression. Therefore, the calculated gene expression can be directly used for comparing the difference of gene expression among samples. Principal coordinate analysis (PCoA) was completed using edgeR.^7^ The differentially expressed genes (DEGs) and transcripts in each sample were determined using the Salmon (version 0.14.1) and DESeq2 (version 1.24.0)^8^ program with default settings. Significant differences in gene expression were evaluated using one-way ANOVA with a set of value of *p*<0.05, and fold-change ≥2. Goatools^9^ is exploited to identify statistically significantly enriched Gene Ontology (GO) term using Fisher’s exact test. The purpose of performing FDR correction is to reduce the Type-1 error by Bonferroni, Holm, BY, BH (multiple hypothesis test method). After multiple testing correction, GO terms with corrected value of *p*≤0.05 are significantly enriched in DEGs. KOBAS 2.0^10^ is exploited to identify statistically significantly enriched pathway using Fisher’s exact test. The purpose of performing FDR correction is to reduce the Type-1 error by Bonferroni, Holm, BY, BH (multiple hypothesis test method). The calculating formula of value of *p* and corrected value of *p* is similar with that in GO analysis. After multiple testing correction, we choose pathways with value of *p*<0.05 that are significantly enriched in DEGs. Functional analyses of the DEGs were performed using the Kyoto Encyclopedia of Genes and Genomes (KEGG), GO, Swiss-Prot (version 2019.7.1), and Pfam (version v32.0) databases.

### Proteomics Analyses of *E. coli* EcNR2 and *E. coli* C321.ΔA

After washing three times with phosphate buffer at 4°C, the pelleted cells were resuspended in 2ml of lysis buffer (20mM HEPES, 10mM NaOH, pH 7.5) containing 150mM NaCl and 1% protease inhibitor cocktail (GE Healthcare, Pittsburgh, United States) and sonicated on ice. After centrifugation at 15,000×*g* for 20min to remove cell debris, the protein samples were precipitated using trichloroacetic acid at a final concentration of 20% (w/v) and washed with ice-cold acetone. After air drying, the precipitated proteins were stored at −80°C until use. Total proteins were analyzed by SDS-PAGE, and the concentrations were determined using the Bradford method with bovine serum albumin as a standard. Before mass spectrometric analysis, the protein samples were dissolved in 8M urea/100mM NH_4_HCO_3_ and subjected to reductive alkylation and trypsin hydrolysis. Three biological replicates were performed for each strain in the proteomic analyses.

The Mascot and Proteome Discoverer software was used to search the raw LC–MS/MS data against the UniProt *E. coli* database. Significant differences in protein expression were evaluated using one-way ANOVA with a set of value of *p*<0.05 and fold-change ≥2. Differentially expressed proteins were analyzed by KEGG pathway analysis and GO analysis using the Swissport database and the Pfam database to identify significantly enriched KEGG pathways and GO functions. Based on the GO and KEGG categories, proteins were classified according to cellular components, molecular functions, and biological processes.

### Plasmid Construction and Mutagenesis

Detailed information on the plasmids used in this study is presented in Table 1. The plasmids pCAS, pTargetF, pULTRA-CNF, and pEVOL-pBpF were obtained from Addgene. The plasmids pBAD24-*GFP* and pET26b-*GFP* were previously used in our laboratory. The gene of a previously reported orthogonal tRNA-synthetase pair that incorporated 5-hydroxytryptophan (5-HTP) into proteins was chemically synthesized and cloned into a pULTRA plasmid to generate the plasmid pULTRA-5HTP. The primers used in this study are listed in Table 2. The primer pairs GFP-Y39TAG-F and GFP-Y39TAG-R, GFP-Y151TAG-F and GFP-Y151TAG-R, and GFP-Y182TAG-F and GFP-Y182TAG-R were used to construct amber mutations at the permissive sites of GFP using the QuikChange site-directed mutagenesis method (Papworth, 1996).

**Table 2:** Primers used in this study.

Primer	Sequence (from 5' to 3')^a^
T7-C321-F	TTCCCCTGATGCTGCCTTACGCGAACGCGAAGTCCGACTCT
T7-C321-down-R	GTGAAACCAGTAACGTTATACGAT
C321-UP-F	TGCCTCTACTGCTGGCGCA
C321-UP-R	TCGGACTTCGCGTTCGCGTAAGGCAGCATCAGGGGAAAACCTTAT
SgRNA	GTGCCCGGCTTCTGACCATG
C321-JD-F	CATGTGCCTTCTTCCGCGTGCA
C321-JD-R	GGCCAGCCACGTTTCTGCGAAA
T7 RNApoly-F	ATGAACACGATTAACATCGCTAAGA
T7 RNApoly-R	TTACGCGAACGCGAAGTCCGACTCT
GFP-Y151TAG-F	CACAATGTA *TAG* ATCATGGCAGACAAACAAAAGAATGGAATCAAAG
GFP-Y151TAG-R	CTGCCATGAT *CTA* TACATTGTGTGAGTTATAGTTGTATTCCAATTTG
GFP-Y39TAG-F	TGCAACA *TAG* GGAAAACTTACCCTTAAATTTATTTGCAC
GFP-Y39TAG-R	GTTTTCC *CTA* TGTTGCATCACCTTCACCCTCTCCACTG
GFP-Y182TAG-F	GCAGACCAT *TAG* CAACAAAATACTCCAATTGGCGATGGC
GFP-Y182TAG-R	TTGTTG *CTA* ATGGTCTGCTAGTTGAACGCTTCCATCTTCAATG

a The underlined sequences indicate mutated codons.

### Integration of T7 RNA Polymerase Into the Genome of *E. coli* C321.ΔA.exp

T7 RNA polymerase was integrated into the genome of *E. coli* C321.ΔA.exp using the CRISPR-Cas9 system (Jiang et al., 2015). The T7 RNA polymerase gene was first amplified by PCR from the genome of *E. coli* BL21(DE3) using the primer T7-C321-F/T7-C321down-R pair, and the homology arms were amplified using the genome of *E. coli* C321.ΔA.exp as a template with the primer C321-UP-F/C321-UP-R pair. The two resulting PCR products were used to amplify the T7 RNA polymerase expression cassettes by overlapping PCR with the primers C321-UP-F/T7-C321down-R. Genome editing experiments were performed as described by Jiang et al. (2015). Briefly, *E. coli* C321.ΔA.exp was first transformed with pCas and induced with arabinose (10mM final concentration) to express recombination-related proteins. After induction, the harvested cells were used to generate competent cells. Then, 100ng of pTarget-*lacZ* plasmid and 400ng of T7 RNA polymerase expression cassette DNA were co-electroporated into competent cells. After recovery at 30°C for 1h, the cells were spread onto LB agar containing kanamycin and spectinomycin and incubated overnight at 30°C. Transformants were identified by colony PCR with the primer C321-JD-F/C321-JD-R pair, and DNA sequencing with the primer T7 RNApoly-F/T7 RNApoly-R pair.

To cure the gene editing-related plasmids, the verified edited colony was inoculated into 5ml of LB medium containing IPTG and kanamycin and cultured at 30°C for 12h. The culture was spread onto LB plates containing kanamycin and incubated at 30°C overnight. Then, the colonies were confirmed to be cured of plasmid pTarget-*lacZ* by determining their sensitivity to spectinomycin. The pCas plasmid was cured by incubating the colonies without plasmid pTarget-*lacZ* in LB medium overnight at 37°C. Finally, *E. coli* C321.ΔA.expT7 with a free plasmid was verified by colony PCR.

### Growth Assay of Different *E. coli* Strains

A single colony of each *E. coli* strain was picked and grown in LB medium at 37°C overnight. The resulting cultures were normalized and diluted to an OD_600_ of 0.05, in 50ml of fresh LB medium. The strains were incubated at 37°C, and the OD_600_ values were measured every 30min for 12h.

### In-Cell Fluorescence Assay

The *E. coli* strains harboring two plasmids, one containing different orthogonal tRNA-synthetase pairs and the other containing GFP variant genes, were grown at 37°C in 50ml of LB supplemented with antibiotics. After the OD_600_ values of the cultures reached 0.4, 1mM of ncAAs was added to the cultures and shaken for another 40min. Then, the inducer IPTG or/and arabinose with final concentrations of 0.4 and 10mM was added to the medium to induce protein expression. After induction at 30°C for 10h, 1ml of the culture was harvested by centrifugation at 8,000×*g* for 10min. The cell pellets were washed three times and suspended in 1ml of phosphate buffer. After normalization, the GFP fluorescence of the cells was measured using a plate reader (E_x_/E_m_=488/513nm). Notably, the cells used to express 5HTP containing proteins were cultured in M9 medium.

### Protein Expression and Purification

Recombinant GFP variants containing different ncAAs were produced in *E. coli* strains, as described above. The harvested cells were suspended in phosphate buffer and sonicated on ice. After centrifugation at 13,000×*g* for 10min at 4°C, the soluble fraction containing the recombinant proteins was recovered and purified using a Ni^2+^-charged chelating Sepharose Fast Flow column (GE Healthcare, Uppsala, Sweden). The purified proteins were analyzed by SDS-PAGE, and the concentrations were measured using the Bradford method with bovine serum albumin as a standard (Bradford, 1976).

### Statistical Analysis

All data in this study were evaluated using one-way ANOVA in SPSS version 20.0. Statistical significance was set at *p*<0.05.

## Results

### Comparative Analyses of the Transcriptome Profiles of *E. coli* C321.ΔA

**Figure 1:**
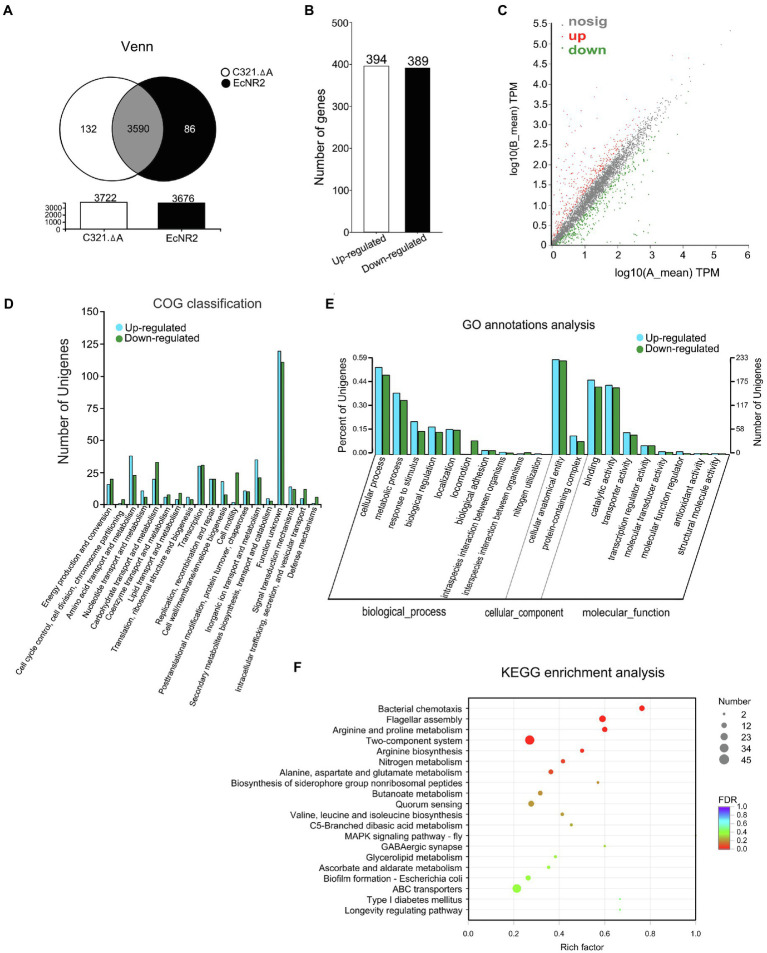
The transcriptome profiles of *Escherichia coli* C321.ΔA and its parent strain. **(A)** A venn diagram of shared and unique of DEGs between *E. coli* C321.ΔA and its parent strain. **(B)** Up- and down-regulated expressed gene count. **(C)** A scatter plot represents the overall changes of the transcriptomic profiles of *E. coli* C321.ΔA. The horizontal and vertical coordinates represent the expression of genes in the two strains. The values of the horizontal and vertical coordinates are logarithmic, with each point representing a specific gene. The red points in the graph indicate significantly up-regulated genes, the green points indicate significantly down-regulated genes, and the gray points are non-significantly different genes. When all genes are mapped, the closer the points are to zero, the lower the expression; those points that deviate more from the diagonal indicate that the gene is more differentially expressed between the two samples. **(D)** Functional classification of the DEGs according to COG. In the horizontal coordinates, the functional classification names were shown. **(E)** Gene Ontology (GO) annotations analyses of the DEGs. **(F)** Kyoto Encyclopedia of Genes and Genomes (KEGG) enrichment analyses of the DEGs. The vertical axis represents the pathway name, and the horizontal axis represents the Rich factor. The size of the dots indicates the number of genes in the pathway, while the color of the dots corresponds to the different FDR-value ranges.

The strain *E. coli* EcNR2 is the parent strain of *E. coli* C321.ΔA (Lajoie et al., 2013). To reveal the potential effects of UAG codon reassignment on *E. coli*, the transcriptomic information of *E. coli* C321.ΔA and *E. coli* EcNR2 was identified and compared using transcript sequencing. After removing the low-quality reads, the number of clean reads obtained from each sample ranged from 31.25 to 35.48 million, with genome mapped ratios from 97.50 to 99.31%. PCA analysis and correlation coefficient analysis revealed that greater variability existed between the two *E. coli* strains than within each biological replicate (Supplementary Figures S1, S2). The results suggested that the replicates in each group had reproducible transcriptome profiles, and UAG codon reassignment could alter the transcriptomic pattern of *E. coli*. The total number of expressed genes in *E. coli* C321.ΔA and *E. coli* EcNR2 was 3,722 and 3,676, respectively (Figure 1A). A total of 132 genes were uniquely expressed in *E. coli* C321.ΔA, whereas 86 genes were uniquely expressed in *E. coli* EcNR2. The threshold for identification of significantly DEGs was set as at least twofold-change and *p*<0.05. Compared with *E. coli* EcNR2, 394 genes were upregulated, and 389 genes were downregulated in *E. coli* C321.ΔA (Figure 1B). Thus, DEGs accounted for approximately 21.04% of the total number of expressed genes. A scatter plot was constructed to represent the overall changes in the DEGs between *E. coli* C321.ΔA and *E. coli* EcNR2 (Figure 1C). Detailed information on the upregulated and downregulated genes, including gene names, fold changes, and function categories, is shown in Supplementary Tables S1 and S2.

A total of 783 DEGs identified from the transcripts were assigned to 19 clusters of orthologous groups (COG; Figure 1D). According to the COG classification analysis, 231 genes were classified into the category “unknown function, [S],” accounting for 29.50% of the total number of DEGs. The function class containing the relatively most significantly up-expressed genes was “Cell wall/membrane/envelope biogenesis, [M],” whereas the function class containing the relatively most significantly down-expressed genes was “Cell motility, [N].”

To further assess the effects of the transcript changes caused by UAG codon reassignment on *E. coli*, we performed GO annotation and KEGG pathway analysis to identify the functional classification of the DEGs. As shown in Figure 1E, GO analysis mapped all of the DEGs into biological processes, cellular components, and molecular functions *via* biological process functional categorization. Within the “biological process” function class, the largest numbers of DEGs were related to the “cellular process” subclass, whereas within the “cellular component” class, the “cellular anatomical entity” subclass contained the most DEGs. Within the “molecular function” function class, the “binding” and “catalytic activity” subclasses had the largest number of DEGs. The metabolic pathways affected by the DEGs were revealed by KEGG pathway analysis. The 20 most abundant metabolic pathways in the DEGs are shown in Figure 1F, and four significantly (*p*<0.05) enriched KEGG pathways were “Flagellar assembly,” “Bacterial chemotaxis,” “Arginine and proline metabolism,” and “Two-component system.” The metabolic pathway “Two-component system” contained the largest number of DEGs when compared with other enriched KEGG pathways.

### Comparative Analyses of the Proteome Profiles of *E. coli* C321.ΔA

**Figure 2:**
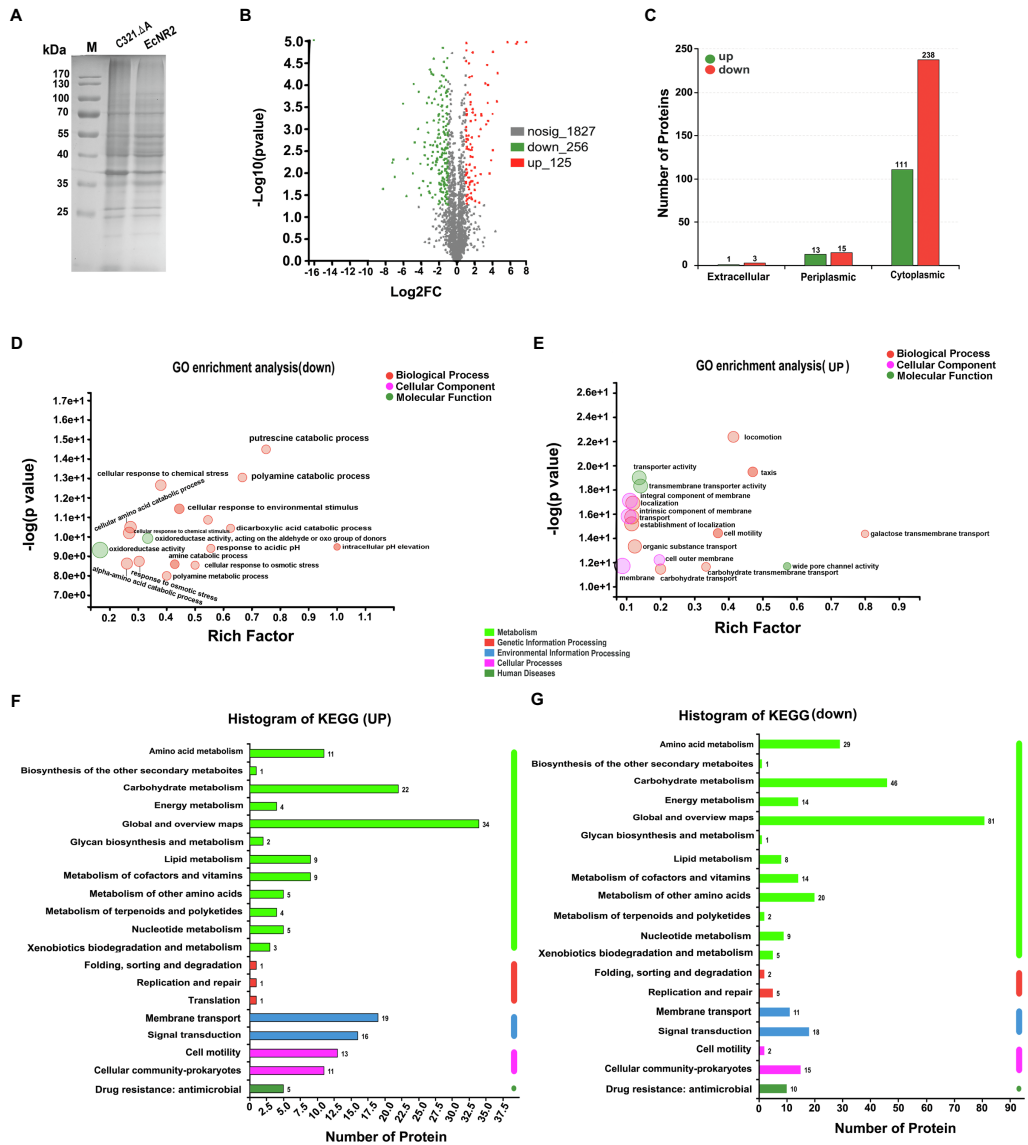
The proteome profiles of *E. coli* C321.ΔA and its parent strain. **(A)** SDS-PAGE analysis of the whole cell proteins of the strain *E. coli* C321.ΔA and its parent strain. **(B)** Differential volcano plot of different expressed proteins. The horizontal coordinate is the value of the fold change in protein expression between the two strains, and the vertical coordinate is the statistical test value of the difference in gene expression. Each point in the graph represents a specific protein, the further to the left and up the more significant the difference in expression. **(C)** Prediction analysis of subcellular localization of the significantly different expressed proteins. **(D,E)** GO enrichment analyses of the different expressed proteins. **(F,G)** KEGG pathways analysis of the different expressed proteins.

To determine the potential effects of UAG codon reassignment on the proteome of *E. coli*, the protein synthesis profiles of *E. coli* C321.ΔA and *E. coli* EcNR2 were identified and compared using label-free proteomic sequencing analysis. The whole cell proteins of *E. coli* C321.ΔA and *E. coli* EcNR2 were first analyzed by SDS-PAGE (Figure 2A). According to the identified proteomic information, the total number of expressed proteins in *E. coli* C321.ΔA and *E. coli* EcNR2 was 2,089 and 2,221, respectively (Supplementary Figure S3). The number of unique proteins identified in *E. coli* C321.ΔA and *E. coli* EcNR2 was 31 and 63, respectively. The threshold for identification of significantly differentially expressed proteins was set as at least twofold-change, and *p*<0.05. The overall significant changes in the proteome of *E. coli* C321.ΔA were represented by a differential volcano plot (Figure 2B). Compared with *E. coli* EcNR2, 256 proteins were downregulated, and 125 proteins were upregulated. Thus, the significantly differentially expressed proteins accounted for roughly 18.24% of the total expressed proteins in *E. coli* C321.ΔA. The locations of the differentially expressed proteins in *E. coli* are shown in Figure 2C, and 91.60% of the identified differentially expressed proteins, including 111 upregulated and 238 downregulated proteins, were cytoplasmic.

The significantly differentially expressed proteins were analyzed by GO enrichment and KEGG pathway analyses. According to GO categorization, the 10 most relatively abundant down-expressed groups were “intracellular pH elevation,” “pH elevation,” “putrescine catabolic process,” “glucarate catabolic process,” “glucarate metabolic process,” “D-glucarate catabolic process,” “D-glucarate metabolic process,” “glycine decarboxylation *via* glycine cleavage system,” “cellular response to acidic pH,” and “polyamine catabolic process,” whereas the top 10 relatively abundant up-expressed groups were “galactose transmembrane transport,” “methylgalactoside transport,” “glycoside transport,” “L-lyxose metabolic process,” “methyl accepting chemotaxis protein complex,” “transmembrane signaling receptor activity,” “porin activity,” “wide pore channel activity,” “hemotaxis,” and “taxis” (Figures 2D,E). As shown in Figure 2F, a large proportion of the upregulated proteins was enriched in seven KEGG pathways, including “Global and overview maps,” “Carbohydrate metabolism,” “Membrane transport,” “Signal transduction,” “Cell motility,” “Cellular community – prokaryotes,” and “Amino acid metabolism.” The down-expressed proteins were abundant in “Global and overview maps,” “Carbohydrate metabolism,” “Amino acid metabolism,” “Metabolism of other amino acids,” “Signal transduction,” “Cellular community – prokaryotes,” “Metabolism of cofactors and vitamins,” “Energy metabolism,” “Membrane transport,” and “Drug resistance: antimicrobial” (Figure 2G).

### Integration of the T7 RNA Polymerase Gene Into the Genome of *E. coli* C321.ΔA. exp

Without the competition of RF1, the orthogonal tRNA-synthetase pair could efficiently decode the amber UAG codon and remarkably improve the yields of ncAA-containing proteins in *E. coli* C321.ΔA (Lajoie et al., 2013). However, the fitness of *E. coli* C321.ΔA is far lower than that of its non-recoded ancestor, which limits its application in many fields, including industrial-scale protein production, metabolic engineering, and live cell imaging (Wannier et al., 2018). Using multiplex genome engineering and predictive modeling, Kuznetsov et al. (2017) recovered the fitness deficit of *E. coli* C321.ΔA and generated the strain *E. coli* C321.ΔA. exp. *Escherichia coli* C321.ΔA. exp showed slightly enhanced ncAA-dependent protein expression. To facilitate the incorporation of ncAAs into proteins using the pET series plasmids in *E. coli* C321.ΔA. exp and further improve the yields of ncAA-containing proteins, we tried to integrate the T7 RNA polymerase gene into the genome using the CRISPR-Cas9 system in this study.

**Figure 3:**
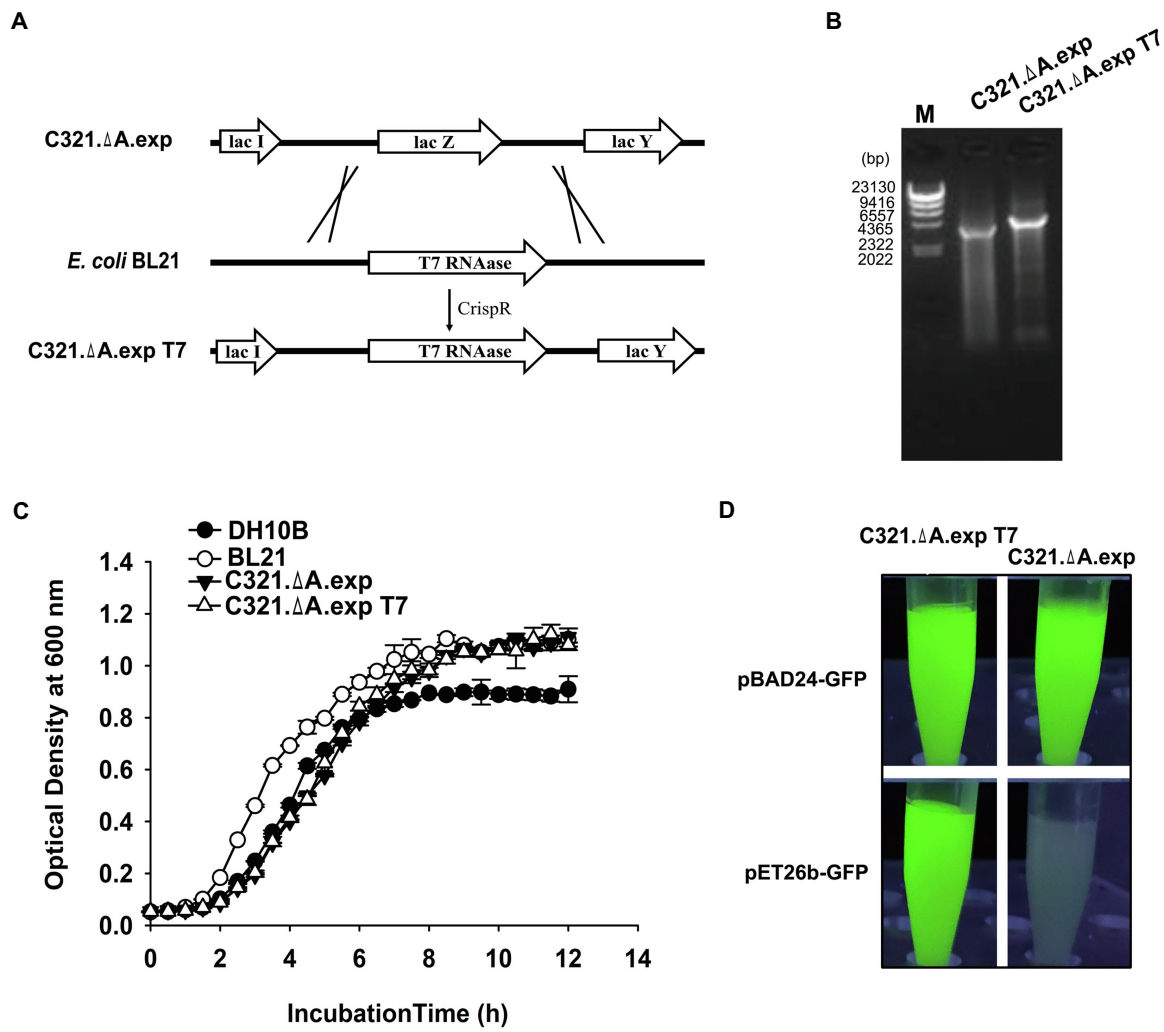
Integration of T7 RNA polymerase gene in *E. coli* C321.ΔA. exp and verification. **(A)** Display of the overall strategy used in this study to integrate T7 RNA polymerase into the genome of *E. coli* C321.ΔA.exp. The crossing lines mean that the LacZ gene in the strain C321. exp is replaced by the T7 RNAase gene amplified from the BL21 genome. **(B)** PCR verification of the successful integration of T7 RNA polymerase in the genome of *E. coli* C321.ΔA.exp. **(C)** Growth curves of the *E. coli* strains used for recombinant protein expression in this study. **(D)** Expression of GFP in *E. coli* C321.ΔA.exp and *E. coli* C321.ΔA.exp T7 with plasmids pBAD24-*GFP* and pET26b-*GFP*.

The T7 RNA polymerase expression cassette was amplified from the *E. coli* BL21(DE3) genome and inserted into the genome of *E. coli* C321.ΔA. exp to replace the *lac Z* gene *via* the CRISPR-Cas9 system, as described in the “Materials and Methods” section (Figure 3A). Genomic PCR screening and sequencing verified that T7 RNA polymerase was integrated into the genome of *E. coli* C321.ΔA. exp (Figure 3B). The T7 RNA polymerase inserted strain was named *E. coli* C321.ΔA. exp T7. We tested whether insertion of the T7 RNA polymerase gene affected the growth of *E. coli* C321.ΔA. exp. According to Figure 3C, the growth rate of *E. coli* C321.ΔA. exp T7 was similar to that of *E. coli* C321.ΔA. exp, indicating that integration of the T7 RNA polymerase gene had limited effects on the strain growth. Next, we investigated whether the inserted T7 RNA polymerase gene could work well in *E. coli* C321.ΔA. exp by performing recombinant protein expression using pET derivative plasmids. As shown in Figure 3D, both *E. coli* C321.ΔA. exp and *E. coli* C321.ΔA. exp T7 could detect strong GFP fluorescence when harboring the pBAD24-*GFP* plasmid after induction with arabinose, but only *E. coli* C321.ΔA.exp T7 showed remarkable GFP fluorescence when both strains harbored the pET26b-*GFP* plasmid after induction with IPTG. Thus, we experimentally verified that pET derivative plasmids could not be used in *E. coli* C321.ΔA.exp directly because of the lack of T7 RNA polymerase, but could work well in our strain *E. coli* C321.ΔA.exp T7.

### The Ability of Incorporation of Different ncAAs at Multiple UAG Sites in *E. coli* C321.ΔA.exp T7

**Figure 4:**
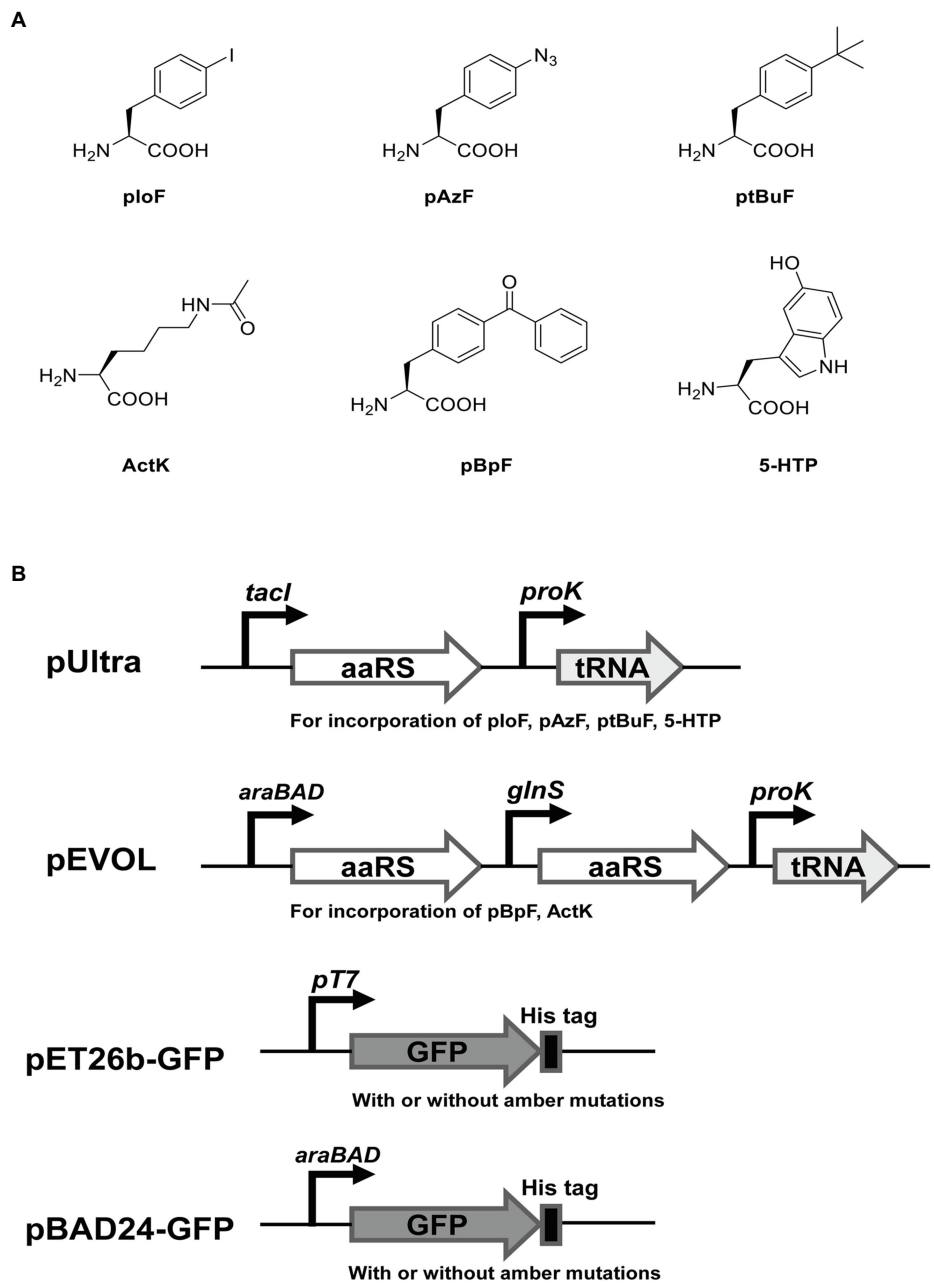
The noncanonical amino acids (ncAAs) and plasmids used in this study for recombinant GFP expression. **(A)** Structures of ncAAs. **(B)** The main features of plasmids.

To test the ability of incorporation of different ncAAs at multiple UAG sites in *E. coli* C321.ΔA.exp T7, we used two widely reported orthogonal plasmids (pEVOL and pUltra derivatives) to incorporate six ncAAs into GFP with amber mutations. For comparison, we also performed the same experiments in *E. coli* BL21(DE3), *E. coli* DH10B, and *E. coli* C321.ΔA.exp. The structures of ncAAs and the main features of the plasmids used in this experiment are shown in Figures 4A,B. According to the growth curves, *E. coli* BL21(DE3) grew faster in the exponential phase than *E. coli* C321.ΔA.exp and *E. coli* C321.ΔA.exp T7, but the same biomass accumulated in the stationary phase. *Escherichia coli* DH10B showed a similar growth rate to *E. coli* C321.ΔA.exp and *E. coli* C321.ΔA.exp T7, but had a slightly lower biomass in the stationary phase (Figure 3C).

**Figure 5:**
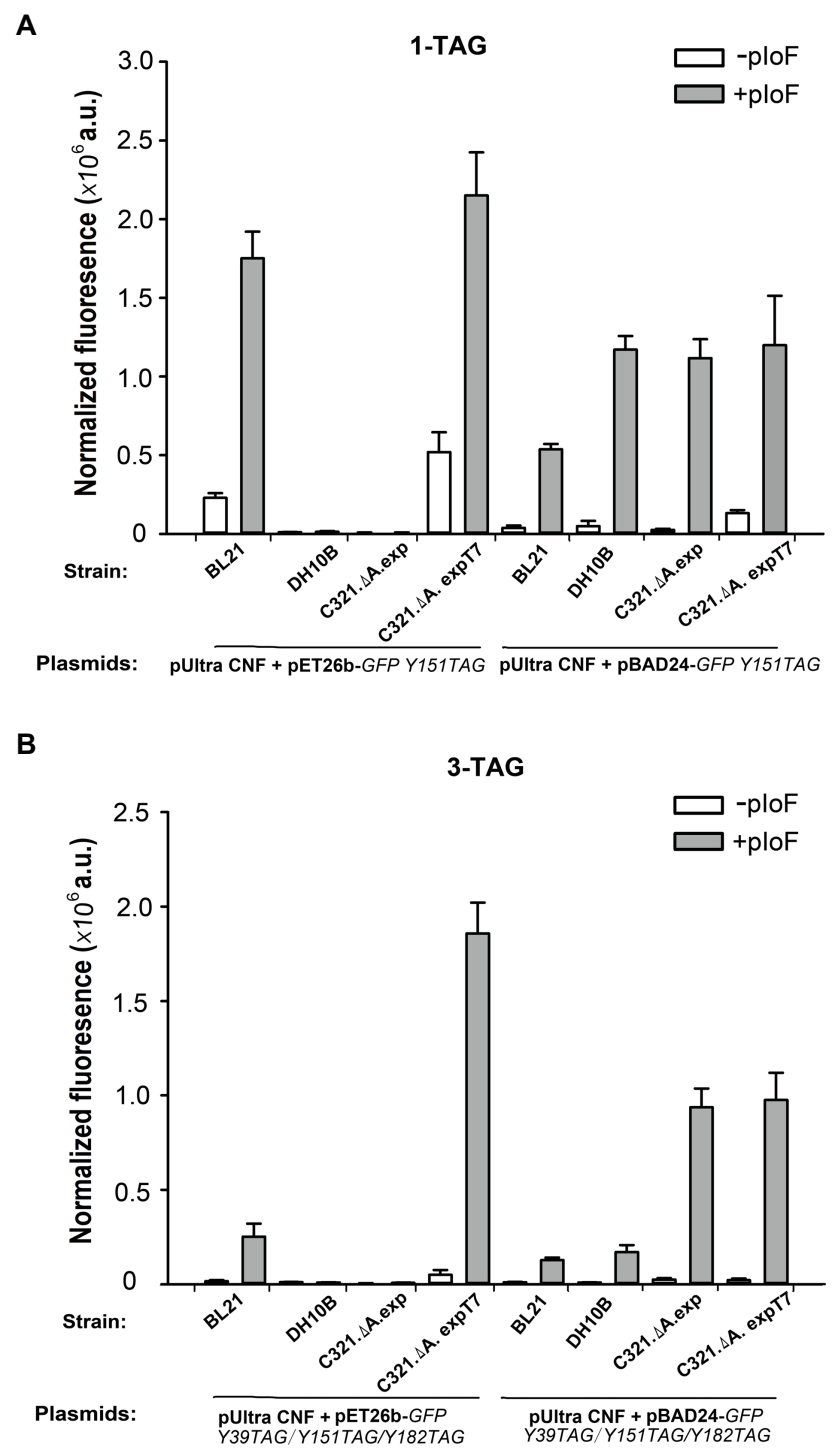
Incorporation of pIoF into GFP containing amber mutations in different strains. **(A)** In-cell GFP fluorescence intensity assay of different strains harboring pUltra-CNF, and pBAD24-*GFPY151TAG* or pET26b-*GFPY151TAG* in the presence or absence of 1mM pIoF in the medium. **(B)** In-cell GFP fluorescence intensity assay of different strains harboring pUltra-CNF, and pBAD24-*GFPY39TAG/Y151TAG/Y182TAG* or pET26b-*GFPY39TAG/Y151TAG/Y182TAG* in the presence or absence of 1mM pIoF in the medium. The values are expressed as means±SD from three independent experiments.

We first compared the GFP expression ability of the four strains when using pIoF as a building block. As shown in Figure 5A, when using pET26b as a vector for expression of the *GFP Y151TAG* gene, only strains *E. coli* BL21(DE3) and *E. coli* C321.ΔA.exp T7 had strong in-cell GFP fluorescence when supplemented with 1mM pIoF. The strains *E. coli* DH10B and *E. coli* C321.ΔA.exp did not show any visible fluorescence due to the lack of T7 RNA polymerase and the inability to use the T7 promoter in the pET26 plasmid. In *E. coli* strain C321.ΔA.exp T7 harboring plasmid pET26b-*GFPY151TAG*, a relatively high GFP fluorescence was also detected in the absence of pIoF. We speculated that the reason for this phenomenon is that UAGs in the GFP gene could be misread by endogenous tRNAs after RF1 removal in *E. coli* C321.ΔA.exp T7, as described by Johnson et al. (2012). When using plasmid pBAD24 as a vector for expression of the *GFP Y151TAG* gene, all four strains showed remarkable in-cell GFP fluorescence in the presence of 1mM pIoF. *Escherichia coli* DH10B showed stronger fluorescence than *E. coli* BL21(DE3), which may be due to the inability of *E. coli* DH10B to catabolize the inducer L-arabinose and could express more intact pIoF-containing GFP variants. *Escherichia coli* BL21(DE3) showed weaker fluorescence than *E. coli* C321.ΔA.exp and *E. coli* C321.ΔA.exp T7 may be attributed to the competition between RF1 and premature translation termination in *E. coli* BL21(DE3). We also determined the ability of the four strains to incorporate pIoF at the three UAG sites in GFP. As shown in Figure 5B, *E. coli* C321.ΔA.exp T7 harboring plasmid pET26b-*GFPY39TAG/Y151TAG/Y182TAG* had stronger fluorescence than the other three strains, indicating that the yields of the pIoF-containing protein could be remarkably improved with the expression of proteins with multiple UAG sites in *E. coli* C321.ΔA.exp T7.

**Figure 6:**
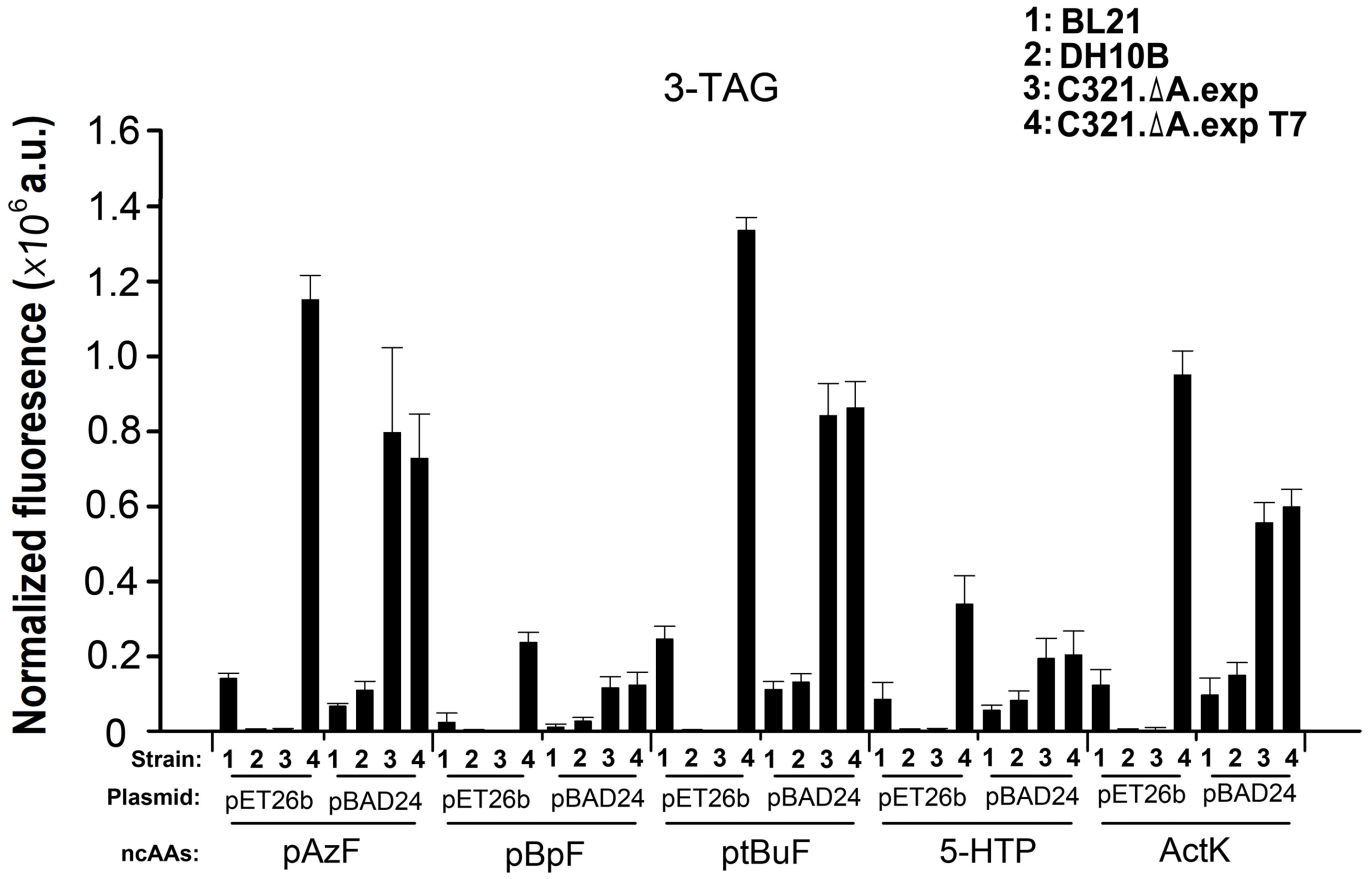
Incorporation of various ncAAs into GFP containing three amber mutations at different positions in different strains. In-cell GFP fluorescence intensity assay of different strains harboring pUltra-CNF, and pBAD24-*GFP* or pET26b-*GFP* containing 3-TAG mutations in the presence or absence of 1mM various ncAAs in the medium. The values are expressed as means±SD from three independent experiments.

Next, we compared the ability of the four strains to incorporate five other ncAAs at three UAG sites in GFP. As shown in Figure 6, regardless of the ncAAs and orthogonal tRNA-synthetase pairs used, *E. coli* C321.ΔA.exp T7 harboring the plasmid pET26b-*GFPY39TAG/Y151TAG/Y182TAG* showed the highest fluorescence among the samples. This result demonstrates that pET derivative plasmids are better than pBAD derivative plasmids in improving the yields of ncAA-containing proteins in *E. coli* C321.ΔA derivative strains. The purified GFP with different ncAAs expressed in *E. coli* C321.ΔA.exp T7 harboring plasmid pET26b-*GFPY39TAG/Y151TAG/Y182TAG* is shown in Supplementary Figure S3. *Escherichia coli* C321.ΔA.exp and *E. coli* C321.ΔA.exp T7 harboring pBAD24-*GFPY39TAG/Y151TAG/Y182TAG* also showed relatively strong in-cell fluorescence, but was lower than that of *E. coli* C321.ΔA.exp T7 harboring the plasmid pET26b-*GFPY39TAG/Y151TAG/Y182TAG*. We speculate that this may be because *E. coli* C321.ΔA.exp and *E. coli* C321.ΔA.exp T7 degrade and reduce the concentration of the inducer L-arabinose during recombinant protein expression, thus, decreasing the yield of the protein products.

## Discussion

The incorporation of ncAAs with different functional groups into proteins has been used in many biological fields, including exploring biological processes, designing fluorescent protein probes, capturing transient protein–protein interactions, enhancing protein drugs, and enzyme-directed evolution (Xiao and Schultz, 2016). Thus, the production of ncAA-containing proteins on an industrial scale is of great significance. In the past few decades, researchers have developed many techniques to incorporate ncAAs into proteins. One such technique is solid-phase peptide synthesis (Kimmerlin and Seebach, 2005). Polypeptide chains no larger than 50 residues can be directly synthesized using canonical amino acids and ncAAs. This method cannot be used to synthesize large proteins (de Graaf et al., 2009). However, this drawback can be partially compromised by some intein-based peptide ligation reactions. Cell-free translation systems can also be used to express proteins with ncAAs (Noren et al., 1989). The key point of this method is the preparation of aminoacylated tRNAs with ncAAs, which can be used by ribosomes to synthesize ncAA-containing proteins. Some ncAAs (structural analogs of canonical amino acids) can be mischarged onto wild-type tRNAs by endogenous corresponding aminoacyl-tRNA synthetases *in vivo* (Link et al., 2003; Hendrickson et al., 2004). In a strain auxotrophic for a certain canonical amino acid, the corresponding ncAA-analog in the medium can be taken by the cells, replacing the canonical amino acid at all sites in the whole proteome of the strain (Ibba and Hennecke, 1995; Sharma et al., 2000). This method may have toxic effects on host cells and restrict the yield of the target protein. Although these methods are useful for incorporating ncAAs into target proteins, they have their own limitations that include not being site-specific, being unsustainable, and only being applicable for canonical amino acid analogs.

Genetic code expansion can overcome most of the aforementioned drawbacks. It can be used to incorporate various ncAAs site-specifically and at multiple sites into target proteins in all living organisms (Chin, 2014). However, due to the competition between RFs and restriction of the endogenous ribosome, the yields of ncAA-containing proteins are usually lower. It has been proven that *E. coli* C321.ΔA and its derivative strains used as expression hosts could significantly improve the yields of ncAA-containing proteins, especially with expressed proteins containing nsAAs at multiple sites (Amiram et al., 2015). To further understand the molecular biology of *E. coli* C321.ΔA, we analyzed the transcriptome and proteome of this strain by high-throughput sequencing and label-free proteomic sequencing, respectively. Our results revealed that both the transcriptome and proteome of *E. coli* C321.ΔA showed significant differences when compared with that of its parent strain *E. coli* EcNR2. Some differentially expressed functions and metabolic pathways were identified in this study. The genes with the function of “Carbohydrate transport and metabolism” and “Cell motility” were significantly downregulated at the transcriptomic level in *E. coli* C321.ΔA. More strikingly, the gene products with the function of “Carbohydrate metabolism,” “Energy metabolism,” and “Amino acid metabolism” were remarkably downregulated at the proteomic level in *E. coli* C321.ΔA. These results indicate that deletion of RF1 and/or UAG codon reassignment may weaken carbohydrate utilization and energy generation in *E. coli*, which may be one of the reasons why *E. coli* C321.ΔA shows a growth defect in the phenotype. However, more studies are needed to strengthen this conclusion, and the molecular mechanism underlying these changes awaits future experimental endeavor.

Although the deletion of RF1 and reassignment of the UAG codon could significantly enhance the production of ncAA-containing proteins, in many cases, the yields of the ncAA-containing proteins are lower than those of the wild-type (Amiram et al., 2015). This phenomenon may be attributed to several factors. The enzymatic activity of the orthogonal tRNA/synthetase pairs usually generated from other living kingdoms may be insufficient in *E. coli*. This problem has been partially overcome by altering the copy number of the orthogonal plasmids, the copy number of orthogonal synthetase/tRNA genes in the plasmids, and the expression strength of the promoters (Young et al., 2010; Chatterjee et al., 2013). Increasing the enzymatic activity of orthogonal translation systems by directed evolution has also been reported to remarkably improve the yields of proteins containing multiple nsAAs (Amiram et al., 2015). The Ef-tu may have suboptimal activity with amino-acylated ncAA-tRNA substrates when delivered into the ribosome, and engineering of Ef-tu to accommodate ncAAs–tRNA substrates could further improve the ncAA-containing protein yields (Park et al., 2011). The development of orthogonal ribosomes is another approach that has been successfully used to enhance the incorporation of unnatural amino acids *in vivo* (Wang et al., 2007). The orthogonal ribosome is generated by directed evolution and can function in parallel with the natural ribosome. This was achieved by designing mutations in 16S rRNA. The mutated 16S rRNA could direct the orthogonal ribosome to the orthogonal messenger RNA containing corresponding complementary mutations in the Shine–Dalgarno sequence. Orthogonal ribosomes can be used to selectively translate orthogonal message RNAs that are not substrates for natural ribosomes.

In genetic code expansion, supplementing ncAAs into the medium is essential to express ncAA-containing proteins during culture fermentation. This would undoubtedly increase the production cost and limit the application of ncAA-containing proteins on an industrial scale, especially when ncAAs are expensive. Autonomous biosynthesis of ncAAs in cells by enzyme evolution and metabolic pathway engineering, and coupling it with genetic code expansion *in situ* could avoid supplementing ncAAs exogenously and significantly save costs. Much progress has been made in this field. By introducing a synthetic pathway from *Streptomyces venezuelae*, *p*-amino-phenylalanine (pAF) has been synthesized in *E. coli* and used as building blocks to produce pAF-containing proteins by genetic code expansion (Chen et al., 2018). 5-HTP has been successfully synthesized in *E. coli* harboring a phenylalanine 4-hydroxylase from *Xanthomonas campestris* and an artificial cofactor regeneration pathway (Chen et al., 2020). The biosynthesized 5-HTP could be genetically incorporated into proteins with high fidelity by genetic code expansion. L-phosphothreonine has been biosynthesized by *Salmonella enterica* kinase in *E. coli* and incorporated into proteins site-specially using a phosphoseryl-tRNA synthetase/tRNA pair (Zhang et al., 2017a). By exogenous feeding of ammonia, catechol, and pyruvate, L-dihydroxyphenylalanine was biosynthesized by a tyrosine phenol-lyase and directly incorporated into proteins by genetic code expansion in *E. coli* (Kim et al., 2018). Similarly, by feeding allyl mercaptan, S-allyl-L-cysteine is biosynthesized from O-acetylserine by PLP-dependent acetylserine sulfhydrylase isozymes of *E. coli* and subsequently incorporated into proteins by an evolved S-allylcysteinyl-tRNA synthetase/tRNA pair (Exner et al., 2017).

Several major challenges need to be addressed to synthesize non-canonical polymers in living cells. First, more aminoacyl-tRNA synthetase tRNA pairs that are orthogonal to not only the natural host synthetases and tRNAs but also to each other need to be developed. Early experiments have demonstrated that the MjTyrRS/tRNA pair and the *P. horikoshii* lysyl-tRNA synthetase/tRNA pair or PylRS/tRNA pair are mutually orthogonal and can be used to incorporate two different ncAAs into a single polypeptide (Wei et al., 2010). Efforts in *de novo* design and generation of multiple mutually orthogonal aaRS/tRNAs have also been reported (Neumann et al., 2010). Italia et al. (2019) developed a set of triply orthogonal pyrrolysyl–tRNA synthetase/tRNA pairs that can be used to decode three distinct non-canonical amino acids in a single polypeptide. Second, new blank codons are required to encode the incorporation of distinct ncAAs into proteins. Since the 61 triplet codons are used in the genome of most organisms for encoding natural amino acids into proteins, stop codons and special quadruplet codons have been used to encode the incorporation of unnatural amino acids before (Chatterjee et al., 2014; Tharp et al., 2021). More blank codons are required to encode non-canonical polymers in living cells. The UAG codon in *E. coli* C321.ΔA was the first blank codon to be assigned for ncAA incorporation. Since then, efforts for synonymous codon replacement, compression, and reassignment in the whole genome have been carried out (Wang et al., 2016). Up to seven codons have been replaced with synonymous alternatives in *E. coli*, and the engineered cells have been used to encode the translation of diverse noncanonical heteropolymers and macrocycles (Ostrov et al., 2016; Robertson et al., 2021). Recently, expanding the genetic alphabet with unnatural base pairs to generate new codons in *E. coli* has been made big breakthroughs by Malyshev et al. (2014). The unnatural codons can be decoded *in vivo* and incorporated ncAAs into proteins (Zhang et al., 2017b). With continuous progress in biochemistry, molecular biology, and chemical biology as described above, we believe that *de novo* design and synthesis of non-canonical polymers in living cells could be achieved in the future.

## Data Availability Statement

The datasets presented in this study can be found in online repositories. The names of the repository/repositories and accession number(s) can be found at: https://www.ncbi.nlm.nih.gov/, PRJNA748036.

## Author Contributions

XGa conceived and supervised the experiments and wrote the initial draft of the manuscript. HY, JZ, FK, XGu, JY, and PX performed the experiments. XGa analyzed the data. LL and QW revised the initial draft of the manuscript. All authors contributed to the article and approved the submitted version.

## Funding

This work was supported by the China Postdoctoral Science Foundation (2020M683364), the Department of Science and Technology of Sichuan Province (2020YJ0129), the Collaborative Fund of the Science and Technology Agency of the Luzhou Government and Southwest Medical University (2020LZXNYDJ29), and the Science Fund Project of the Southwest Medical University (2019ZQN024 and 2020ZRQNA007).

## Conflict of Interest

The authors declare that the research was conducted in the absence of any commercial or financial relationships that could be construed as a potential conflict of interest.

## Publisher’s Note

All claims expressed in this article are solely those of the authors and do not necessarily represent those of their affiliated organizations, or those of the publisher, the editors and the reviewers. Any product that may be evaluated in this article, or claim that may be made by its manufacturer, is not guaranteed or endorsed by the publisher.

## Footnotes

^1^ https://github.com/jstjohn/SeqPrep

^2^ https://www.ncbi.nlm.nih.gov/nuccore/CP006698.1

^3^ http://bowtie-bio.sourceforge.net/bowtie2/index.shtml

^4^ http://rfam.xfam.org/

^5^ www.majorbio.com

^6^ http://deweylab.github.io/RSEM/

^7^ http://www.bioconductor.org/packages/2.12/bioc/html/edgeR.html

^8^ http://bioconductor.org/packages/release/bioc/html/DESeq2.html

^9^ https://github.com/tanghaibao/GOatools

^10^ http://kobas.cbi.pku.edu.cn/home.do
