# Supplementary material for: Comparative Analyses of the Transcriptome and Proteome of Escherichia coli C321.△A and Further Improving Its Noncanonical Amino Acids Containing Protein Expression Ability by Integration of T7 RNA Polymerase
Source: Front Microbiol. 2021 Sep 29;12:744284. doi: 10.3389/fmicb.2021.744284 (PMC8511705; doi:10.3389/fmicb.2021.744284)
Supplement: Supplementary file 1 [file Data_Sheet_1.docx]

**Table S1. The information of down-regulated genes in *E.coli* C321.ΔA**

| Gene ID | Gene name | Fold Change | COG ID | COG_Functional Categories |
| --- | --- | --- | --- | --- |
| N840_3642 | lyxK | 0.003 | COG1070 | G:Carbohydrate transport and metabolism |
| N840_3637 | yiaK | 0.002 | COG2055 | E:Amino acid transport and metabolism |
| N840_3641 | yiaO | 0.001 | COG1638 | G:Carbohydrate transport and metabolism |
| N840_4328 | bdcA | 0.002 | COG1028 | F:Nucleotide transport and metabolism |
| N840_3645 | yiaS | 0.006 | COG0235 | G:Carbohydrate transport and metabolism |
| N840_4327 | yjgH | 0.018 | COG0251 | F:Nucleotide transport and metabolism |
| N840_3644 | yiaR | 0.002 | COG3623 | G:Carbohydrate transport and metabolism |
| N840_0047 | folA | 0.062 | COG0262 | H:Coenzyme transport and metabolism |
| N840_2196 | mglB | 0.05 | COG1879 | G:Carbohydrate transport and metabolism |
| N840_1212 | dhaK | 0.068 | COG2376 | G:Carbohydrate transport and metabolism |
| N840_1121 | bhsA | 0.079 | ENOG4111Y56 | M:Cell wall/membrane/envelope biogenesis |
| N840_2194 | mglC | 0.056 | COG4211 | G:Carbohydrate transport and metabolism |
| N840_2195 | mglA | 0.051 | COG1129 | G:Carbohydrate transport and metabolism |
| N840_3643 | yiaQ | 0.002 | COG0269 | G:Carbohydrate transport and metabolism |
| N840_1210 | dhaM | 0.086 | COG1925 | G:Carbohydrate transport and metabolism |
| N840_3640 | yiaN | 0.003 | COG1593 | G:Carbohydrate transport and metabolism |
| N840_3436 | yhfZ | 0.076 | ENOG410YBQW | S:Function unknown |
| N840_3149 | mzrA | 0.085 | ENOG4112345 | S:Function unknown |
| N840_1211 | dhaL | 0.098 | COG2376 | G:Carbohydrate transport and metabolism |
| N840_1233 | chaA | 0.102 | COG0387 | P:Inorganic ion transport and metabolism |
| N840_2993 | speB | 0.197 | COG0010 | E:Amino acid transport and metabolism |
| N840_3914 | fre | 0.165 | COG0543 | C:Energy production and conversion |
| N840_1465 | yncJ | 0.053 | ENOG41120CN | S:Function unknown |
| N840_3148 | yqjA | 0.18 | COG0586 | G:Carbohydrate transport and metabolism |
| N840_1884 | yobB | 0.17 | COG0388 | L:Replication, recombination and repair |
| N840_0014 | insL1 | 0.137 | COG3385 | L:Replication, recombination and repair |
| N840_1941 | ftnB | 0.221 | COG1528 | P:Inorganic ion transport and metabolism |
| N840_1878 | ryeA | 0.052 | ------ | ------ |
| N840_2998 | galP | 0.17 | ENOG410XNQK | H:Coenzyme transport and metabolism |
| N840_1887 | yebE | 0.147 | COG2979 | S:Function unknown |
| N840_2053 | yeeD | 0.1 | COG0425 | L:Replication, recombination and repair |
| N840_1868 | htpX | 0.225 | COG0501 | P:Inorganic ion transport and metabolism |
| N840_2197 | galS | 0.146 | COG1609 | G:Carbohydrate transport and metabolism |
| N840_1619 | ynfF | 0.189 | COG1104 | S:Function unknown |
| N840_2157 | yehD | 0.069 | ENOG41122IH | S:Function unknown |
| N840_1064 | yceI | 0.132 | COG2353 | S:Function unknown |
| N840_1885 | exoX | 0.249 | COG0847 | L:Replication, recombination and repair |
| N840_1620 | ynfG | 0.181 | COG0437 | S:Function unknown |
| N840_3529 | nikA | 0.113 | COG0747 | H:Coenzyme transport and metabolism |
| N840_1839 | yeaV | 0.049 | COG1292 | K:Transcription |
| N840_4013 | katG | 0.201 | COG0376 | P:Inorganic ion transport and metabolism |
| N840_2785 | mutS | 0 | ENOG41128K4 | S:Function unknown |
| N840_1223 | prmC | 0.198 | COG2890 | J:Translation, ribosomal structure and biogenesis |
| N840_1526 | ydeN | 0.128 | COG3119 | P:Inorganic ion transport and metabolism |
| N840_0226 | dinJ | 0.31 | COG3077 | L:Replication, recombination and repair |
| N840_3435 | yhfY | 0.104 | ENOG4111VUY | S:Function unknown |
| N840_1120 | ycfQ | 0.221 | ENOG41122NH | K:Transcription; |
| N840_3636 | yiaJ | 0.134 | COG1414 | E:Amino acid transport and metabolism |
| N840_3532 | nikD | 0.118 | COG0444 | H:Coenzyme transport and metabolism |
| N840_3930 | dsbA | 0.333 | COG0526 | S:Function unknown |
| N840_1689 | sodB | 0.331 | COG0605 | P:Inorganic ion transport and metabolism |
| N840_1621 | ynfH | 0.201 | COG3302 | S:Function unknown |
| N840_3227 | secG | 0.352 | COG1314 | U:Intracellular trafficking, secretion, and vesicular transport |
| N840_3822 | rbsA | 0.228 | COG1129 | G:Carbohydrate transport and metabolism |
| N840_3780 | tnaC | 0.257 | ------ | ------ |
| N840_3639 | yiaM | 0.001 | COG3090 | G:Carbohydrate transport and metabolism |
| N840_1787 | ydjY | 0.175 | ENOG41100DI | S:Function unknown |
| N840_2546 | focB | 0.074 | COG2116 | K:Transcription |
| N840_1707 | ydhV | 0.193 | COG2414 | C:Energy production and conversion |
| N840_3012 | ansB | 0.258 | COG0252 | S:Function unknown |
| N840_1701 | ydhR | 0.304 | ENOG4111VBS | S:Function unknown |
| N840_1945 | ftnA | 0.187 | COG1528 | S:Function unknown |
| N840_1779 | spy | 0.175 | ENOG410YS8I | S:Function unknown |
| N840_1632 | mdtJ | 0.237 | COG2076 | O:Posttranslational modification, protein turnover, chaperones |
| N840_4329 | bdcR | 0.22 | ENOG4111MNX | K:Transcription |
| N840_1963 | fliC | 0.197 | COG1344 | K:Transcription |
| N840_0477 | recR | 0.334 | COG0353 | L:Replication, recombination and repair |
| N840_2252 | napA | 0.271 | COG0243 | C:Energy production and conversion |
| N840_4308 | ytfR | 0.366 | COG1129 | G:Carbohydrate transport and metabolism |
| N840_3840 | ilvG | 0.254 | COG0028 | O:Posttranslational modification, protein turnover, chaperones |
| N840_1502 | fdnG | 0.243 | COG0243; | E:Amino acid transport and metabolism |
| N840_3985 | cpxP | 0.381 | COG3678 | S:Function unknown |
| N840_3638 | yiaL | 0.001 | COG2731 | E:Amino acid transport and metabolism |
| N840_1086 | flgG | 0.11 | COG4786 | N:Cell motility |
| N840_3530 | nikB | 0.067 | COG0601 | H:Coenzyme transport and metabolism |
| N840_2995 | yqgB | 0.275 | ENOG410Y7K0 | S:Function unknown |
| N840_1294 | yciX | 0.281 | ------ | ------ |
| N840_1453 | opgD | 0.423 | COG3131 | S:Function unknown |
| N840_0412 | yajC | 0.406 | COG1862 | U:Intracellular trafficking, secretion, and vesicular transport |
| N840_1618 | ynfE | 0.299 | COG1104 | S:Function unknown; |
| N840_0501 | ybbA | 0.352 | COG4181 | Q:Secondary metabolites biosynthesis, transport and catabolism |
| N840_3821 | rbsD | 0.313 | COG1869 | G:Carbohydrate transport and metabolism |
| N840_4417 | yjiL | 0.325 | COG1924 | I:Lipid transport and metabolism |
| N840_4341 | yjgR | 0.392 | COG0433 | S:Function unknown |
| N840_0613 | cstA | 0.34 | COG1966 | T:Signal transduction mechanisms |
| N840_0938 | ompF | 0.292 | COG3203 | E:Amino acid transport and metabolism |
| N840_2338 | lrhA | 0.385 | ENOG410XR9D | K:Transcription |
| N840_0331 | yahF | 0.159 | COG0074 | C:Energy production and conversion |
| N840_3533 | nikE | 0.093 | COG1124 | H:Coenzyme transport and metabolism |
| N840_0817 | glnQ | 0.375 | COG1126 | S:Function unknown |
| N840_2734 | ygaH | 0.158 | ENOG4111UT4 | S:Function unknown |
| N840_1083 | flgD | 0.077 | COG1843 | N:Cell motility |
| N840_2295 | rhmD | 0.305 | COG4948 | G:Carbohydrate transport and metabolism |
| N840_1940 | araF | 0.311 | COG1879 | G:Carbohydrate transport and metabolism |
| N840_2969 | ibsC | 0.206 | ------ | ------ |
| N840_0411 | tgt | 0.455 | COG0343 | J:Translation, ribosomal structure and biogenesis |
| N840_2134 | gatR | 0.339 | COG2963 | L:Replication, recombination and repair; |
| N840_0818 | glnP | 0.353 | COG0765 | S:Function unknown |
| N840_0474 | apt | 0.403 | COG0503 | S:Function unknown |
| N840_3531 | nikC | 0.102 | COG1173 | H:Coenzyme transport and metabolism |
| N840_4309 | ytfT | 0.353 | ENOG410XPE9 | S:Function unknown |
| N840_3513 | livJ | 0.304 | COG0683 | S:Function unknown |
| N840_4416 | yjiK | 0.226 | COG3204 | S:Function unknown |
| N840_2155 | yehB | 0.123 | COG3188 | S:Function unknown |
| N840_1930 | cheA | 0.235 | COG0643 | T:Signal transduction mechanisms |
| N840_4307 | ytfQ | 0.341 | ENOG410XPC7 | G:Carbohydrate transport and metabolism |
| N840_0112 | aceF | 0.397 | COG0508 | C:Energy production and conversion |
| N840_0614 | ybdD | 0.4 | COG2879 | S:Function unknown |
| N840_4386 | yjhY | 0.34 | ------ | ------ |
| N840_1122 | ycfS | 0.318 | COG1376 | M:Cell wall/membrane/envelope biogenesis |
| N840_1448 | mokB | 0.387 | ------ | ------ |
| N840_0159 | cdaR | 0.304 | COG3835 | K:Transcription |
| N840_3782 | tnaB | 0.266 | COG0814 | E:Amino acid transport and metabolism |
| N840_3983 | cpxA | 0.422 | ENOG410XTWD | S:Function unknown |
| N840_2254 | napF | 0.204 | COG1145 | P:Inorganic ion transport and metabolism |
| N840_3434 | yhfX | 0.092 | COG3457 | E:Amino acid transport and metabolism |
| N840_4323 | ridA | 0.444 | COG0251 | P:Inorganic ion transport and metabolism |
| N840_3984 | cpxR | 0.452 | COG0745 | S:Function unknown |
| N840_2256 | eco | 0.403 | COG4574 | S:Function unknown |
| N840_2137 | gatD | 0.44 | COG1063 | L:Replication, recombination and repair |
| N840_0502 | ybbP | 0.382 | COG3127 | Q:Secondary metabolites biosynthesis, transport and catabolism |
| N840_1708 | ydhY | 0.219 | COG0437 | C:Energy production and conversion |
| N840_1490 | pptA | 0.321 | COG1942 | S:Function unknown |
| N840_2269 | atoD | 0.052 | COG1788 | I:Lipid transport and metabolism |
| N840_1927 | tap | 0.179 | COG0840 | T:Signal transduction mechanisms |
| N840_3928 | yihD | 0.391 | COG3084 | S:Function unknown |
| N840_0460 | ffs | 0.194 | ------ | ------ |
| N840_2706 | ypjA | 0.267 | ENOG4110GG4 | U:Intracellular trafficking, secretion, and vesicular transport |
| N840_3781 | tnaA | 0.357 | COG3033 | E:Amino acid transport and metabolism |
| N840_0332 | yahG | 0.181 | ENOG410XPNV | S:Function unknown |
| N840_2658 | pheA | 0.395 | COG1605 | E:Amino acid transport and metabolism |
| N840_0754 | pal | 0.492 | COG2885 | M:Cell wall/membrane/envelope biogenesis |
| N840_4326 | pyrL | 0.326 | ENOG410Z990 | F:Nucleotide transport and metabolism; |
| N840_0111 | aceE | 0.463 | COG2609 | C:Energy production and conversion |
| N840_1826 | yeaM | 0.385 | ENOG410XQRW | K:Transcription |
| N840_1928 | tar | 0.175 | COG0840 | T:Signal transduction mechanisms |
| N840_4476 | slt | 0.487 | COG0741 | H:Coenzyme transport and metabolism |
| N840_1194 | hlyE | 0.199 | ENOG410YAUV | D:Cell cycle control, cell division, chromosome partitioning |
| N840_0966 | ompA | 0.426 | COG2885 | S:Function unknown |
| N840_2250 | napH | 0.184 | COG0348 | C:Energy production and conversion |
| N840_2733 | ygaZ | 0.378 | COG1296 | E:Amino acid transport and metabolism |
| N840_2520 | aegA | 0.36 | COG1142 | C:Energy production and conversion |
| N840_0029 | rihC | 0.42 | COG1957 | F:Nucleotide transport and metabolism |
| N840_2400 | gtrS | 0.21 | ------ | ------ |
| N840_0283 | yagJ | 0.271 | ENOG410ZVHQ | V:Defense mechanisms |
| N840_0744 | mngB | 0.393 | COG0383 | G:Carbohydrate transport and metabolism |
| N840_2522 | acrD | 0.405 | COG0841 | C:Energy production and conversion |
| N840_0905 | dmsC | 0.321 | COG3302 | S:Function unknown |
| N840_3635 | ysaA | 0.307 | COG1142 | E:Amino acid transport and metabolism |
| N840_4433 | mrr | 0.405 | COG1715 | V:Defense mechanisms |
| N840_3929 | rdoA | 0.435 | COG2334 | S:Function unknown |
| N840_1931 | motB | 0.161 | COG1360 | T:Signal transduction mechanisms |
| N840_4109 | lamB | 0.355 | COG4580 | G:Carbohydrate transport and metabolism |
| N840_4096 | lysC | 0.228 | COG0527 | S:Function unknown |
| N840_4196 | melB | 0.364 | COG2211 | K:Transcription |
| N840_3020 | speC | 0.399 | COG1982 | P:Inorganic ion transport and metabolism |
| N840_0225 | yafQ | 0.317 | COG3041 | S:Function unknown |
| N840_4363 | yjhV | 0.09 | ------ | ------ |
| N840_3125 | aer | 0.441 | COG0840 | K:Transcription |
| N840_3208 | yhbU | 0.39 | COG0826 | I:Lipid transport and metabolism |
| N840_0210 | gloB | 0.41 | COG0491 | S:Function unknown |
| N840_4136 | soxS | 0.275 | ENOG4111IXA | K:Transcription |
| N840_0904 | dmsB | 0.392 | COG0437 | C:Energy production and conversion |
| N840_1854 | yoaE | 0.462 | COG0861 | P:Inorganic ion transport and metabolism |
| N840_3433 | yhfW | 0.176 | COG1015 | G:Carbohydrate transport and metabolism |
| N840_2140 | gatA | 0.478 | COG1762 | G:Carbohydrate transport and metabolism |
| N840_3810 | atpE | 0.434 | COG0636 | C:Energy production and conversion |
| N840_0362 | mhpT | 0.339 | ENOG410YW8U | G:Carbohydrate transport and metabolism |
| N840_0819 | glnH | 0.485 | COG0834 | S:Function unknown |
| N840_1085 | flgF | 0.084 | COG4787 | N:Cell motility |
| N840_4298 | ytfK | 0.411 | ENOG4111TN3 | S:Function unknown |
| N840_0115 | acnB | 0.491 | COG1049 | C:Energy production and conversion |
| N840_0800 | ybhR | 0.413 | COG0842 | S:Function unknown |
| N840_2347 | yfcC | 0.267 | COG1288 | S:Function unknown |
| N840_1840 | yeaW | 0.299 | COG4638 | P:Inorganic ion transport and metabolism |
| N840_4487 | yjtD | 0.367 | COG0565 | S:Function unknown |
| N840_3863 | wecF | 0.443 | ENOG410XT9P | M:Cell wall/membrane/envelope biogenesis |
| N840_3628 | xylF | 0.409 | COG4213 | G:Carbohydrate transport and metabolism |
| N840_4228 | sugE | 0.446 | COG2076 | S:Function unknown |
| N840_2704 | ypjF | 0.107 | ENOG4111PBJ | S:Function unknown |
| N840_4360 | insO | 0.349 | COG2826 | L:Replication, recombination and repair |
| N840_1082 | flgC | 0.062 | COG1558 | N:Cell motility |
| N840_0870 | artM | 0.445 | COG4160 | E:Amino acid transport and metabolism |
| N840_3048 | hybD | 0.414 | COG0680 | O:Posttranslational modification, protein turnover, chaperones |
| N840_2055 | plaP | 0.405 | COG0531 | L:Replication, recombination and repair |
| N840_0438 | ampG | 0.445 | COG0477 | C:Energy production and conversion |
| N840_2592 | hcaR | 0.491 | ENOG4111IYC | K:Transcription |
| N840_4195 | melA | 0.495 | COG1486 | K:Transcription |
| N840_0560 | nmpC | 0.456 | COG3039 | L:Replication, recombination and repair |
| N840_0801 | ybhS | 0.431 | COG0842 | S:Function unknown |
| N840_4303 | ytfP | 0.488 | COG2105 | S:Function unknown |
| N840_1363 | mcaS | 0.301 | ------ | ------ |
| N840_3049 | hybC | 0.477 | COG0374 | O:Posttranslational modification, protein turnover, chaperones |
| N840_1976 | fliF | 0.101 | COG1766 | N:Cell motility |
| N840_2897 | ygeA | 0.484 | COG1794 | E:Amino acid transport and metabolism |
| N840_1981 | fliK | 0.238 | COG3144 | N:Cell motility |
| N840_1065 | yceJ | 0.344 | COG3038 | C:Energy production and conversion |
| N840_4484 | creD | 0.345 | COG4452 | V:Defense mechanisms |
| N840_0950 | elfG | 0.291 | COG3539 | N:Cell motility;U:Intracellular trafficking, secretion, and vesicular transport |
| N840_1649 | uidA | 0.436 | COG3250 | U:Intracellular trafficking, secretion, and vesicular transport |
| N840_4304 | chpS | 0.346 | COG2336 | S:Function unknown;K:Transcription |
| N840_1788 | ydjZ | 0.355 | COG0398 | S:Function unknown |
| N840_2576 | ryfA | 0.444 | ------ | ------ |
| N840_1790 | ynjB | 0.488 | COG4134 | S:Function unknown |
| N840_4424 | yjiT | 0.383 | ENOG4111M9Y | S:Function unknown |
| N840_0132 | yadC | 0.254 | ENOG4111X8M | U:Intracellular trafficking, secretion, and vesicular transport |
| N840_2524 | yffB | 0.447 | COG1393 | P:Inorganic ion transport and metabolism |
| N840_3432 | php | 0.137 | COG1735 | S:Function unknown |
| N840_4310 | yjfF | 0.413 | ENOG410XR1U | G:Carbohydrate transport and metabolism |
| N840_2117 | sibA | 0.342 | ------ | ------ |
| N840_3141 | alx | 0.437 | COG0861 | S:Function unknown |
| N840_1081 | flgB | 0.038 | COG1815 | N:Cell motility |
| N840_0520 | ylbA | 0.373 | COG3257 | S:Function unknown |
| N840_1837 | dmlR | 0.463 | ENOG410XNQA | K:Transcription |
| N840_1983 | fliM | 0.152 | COG1868 | N:Cell motility |
| N840_1084 | flgE | 0.092 | COG1749 | N:Cell motility |
| N840_2249 | napB | 0.292 | COG3043 | C:Energy production and conversion |
| N840_1447 | hokB | 0.378 | ------ | ------ |
| N840_4349 | intB | 0.483 | COG0582 | K:Transcription |
| N840_2735 | mprA | 0.458 | COG1846 | S:Function unknown |
| N840_1962 | fliA | 0.105 | COG1191 | K:Transcription |
| N840_4395 | fimB | 0.263 | ENOG410YF4U | L:Replication, recombination and repair |
| N840_4152 | gltP | 0.487 | COG1301 | O:Posttranslational modification, protein turnover, chaperones |
| N840_1206 | ycgR | 0.195 | COG5581 | M:Cell wall/membrane/envelope biogenesis |
| N840_1593 | hokD | 0.483 | ------ | ------ |
| N840_1580 | gnsB | 0.41 | ENOG410Y8R8 | S:Function unknown |
| N840_4330 | tabA | 0.404 | COG2731 | K:Transcription |
| N840_1595 | relB | 0.393 | COG3077 | D:Cell cycle control, cell division, chromosome partitioning; |
| N840_2552 | upp | 0.449 | COG0035 | F:Nucleotide transport and metabolism |
| N840_0110 | pdhR | 0.482 | COG2186 | K:Transcription |
| N840_1933 | flhC | 0.497 | ENOG410XXBZ | K:Transcription |
| N840_1601 | dicA | 0.488 | COG1396 | K:Transcription |
| N840_3574 | yhjA | 0.317 | COG1858 | P:Inorganic ion transport and metabolism |
| N840_3883 | xerC | 0.5 | COG4973 | L:Replication, recombination and repair |
| N840_0324 | betI | 0.492 | COG1309 | K:Transcription |
| N840_1570 | ydfZ | 0.355 | ENOG41126WX | S:Function unknown |
| N840_3056 | yqhA | 0.499 | COG2862 | S:Function unknown |
| N840_1929 | cheW | 0.302 | COG0835 | N:Cell motility;T:Signal transduction mechanisms |
| N840_0330 | yahE | 0.264 | ENOG4111W16 | S:Function unknown |
| N840_1558 | marA | 0.378 | ENOG4111IUP | K:Transcription |
| N840_3783 | mdtL | 0.406 | ENOG410YI0P | V:Defense mechanisms |
| N840_1786 | ydjX | 0.419 | COG0398 | S:Function unknown |
| N840_4437 | tsr | 0.424 | COG0840 | V:Defense mechanisms |
| N840_0481 | aes | 0.46 | COG0657 | I:Lipid transport and metabolism |
| N840_3647 | ysaD | 0.004 | COG3533 | S:Function unknown |
| N840_3836 | yifE | 0.453 | COG3085 | S:Function unknown |
| N840_1594 | relE | 0.44 | COG2026 | D:Cell cycle control, cell division, chromosome partitioning |
| N840_0076 | leuO | 0.323 | ENOG4110BBF | K:Transcription |
| N840_1087 | flgH | 0.189 | COG2063 | N:Cell motility |
| N840_1706 | ydhW | 0.366 | ENOG4111TJ2 | S:Function unknown |
| N840_2272 | atoB | 0.247 | COG0183 | I:Lipid transport and metabolism |
| N840_3316 | yhdU | 0.135 | ENOG4112BHE | S:Function unknown |
| N840_2271 | atoE | 0.19 | COG2031 | I:Lipid transport and metabolism |
| N840_3157 | yhaI | 0.199 | COG3152 | S:Function unknown |
| N840_1859 | mntP | 0.39 | COG1971 | S:Function unknown |
| N840_2292 | ypaA | 0.409 | COG5464 | E:Amino acid transport and metabolism |
| N840_1791 | ynjC | 0.471 | COG4135 | S:Function unknown |
| N840_3209 | yhbV | 0.413 | COG0826 | I:Lipid transport and metabolism |
| N840_1224 | ychQ | 0.488 | COG3094 | S:Function unknown |
| N840_1982 | fliL | 0.112 | COG1580 | N:Cell motility |
| N840_2999 | yggI | 0.307 | COG3091 | S:Function unknown |
| N840_2130 | ogrK | 0.388 | ENOG41128D7 | K:Transcription |
| N840_3789 | cbrB | 0.365 | ENOG410Y18Y | S:Function unknown |
| N840_2786 | pphB | 0.177 | COG0639 | S:Function unknown |
| N840_2501 | yffR | 0.452 | ------ | ------ |
| N840_3136 | higB | 0.332 | COG4680 | S:Function unknown |
| N840_1926 | cheR | 0.234 | ENOG410XNMH | T:Signal transduction mechanisms |
| N840_2251 | napG | 0.295 | COG1145 | C:Energy production and conversion |
| N840_0333 | yahI | 0.336 | COG0549 | E:Amino acid transport and metabolism |
| N840_0704 | speF | 0.244 | COG1982 | S:Function unknown |
| N840_4361 | insI1 | 0.477 | COG2826 | L:Replication, recombination and repair |
| N840_1088 | flgI | 0.242 | COG1706 | N:Cell motility |
| N840_3045 | hybG | 0.417 | COG0298 | E:Amino acid transport and metabolism |
| N840_0282 | insA | 0.006 | COG3677 | L:Replication, recombination and repair |
| N840_1446 | ydcA | 0.5 | ENOG4111SBQ | S:Function unknown |
| N840_1970 | yedF | 0.421 | COG0425 | O:Posttranslational modification, protein turnover, chaperones |
| N840_1597 | flxA | 0.363 | ------ | ------ |
| N840_2416 | dsdX | 0.392 | COG2610 | E:Amino acid transport and metabolism; |
| N840_3595 | ldrD | 0.436 | ENOG410Y4X1 | S:Function unknown |
| N840_2426 | yfdX | 0.192 | ENOG4111IX2 | S:Function unknown |
| N840_0955 | zapC | 0.48 | ENOG410YF51 | D:Cell cycle control, cell division, chromosome partitioning |
| N840_2156 | yehC | 0.059 | COG3121 | S:Function unknown |
| N840_1631 | mdtI | 0.466 | COG2076 | O:Posttranslational modification, protein turnover, chaperones |
| N840_1189 | ycgJ | 0.494 | ENOG4111XFY | S:Function unknown |
| N840_2056 | yoeI | 0.329 | ------ | ------ |
| N840_1437 | ynbC | 0.443 | COG2267 | I:Lipid transport and metabolism |
| N840_0059 | araD | 0.401 | COG0235 | G:Carbohydrate transport and metabolism |
| N840_0270 | insI1 | 0.471 | COG2826 | L:Replication, recombination and repair |
| N840_2705 | psaA | 0.462 | ------ | ------ |
| N840_1923 | cheZ | 0.391 | COG3143 | T:Signal transduction mechanisms |
| N840_2291 | yfaD | 0.478 | COG5464 | E:Amino acid transport and metabolism |
| N840_3430 | yhfT | 0.308 | ENOG410XRS1 | S:Function unknown |
| N840_2557 | yfgF | 0.384 | ENOG410XNMH | F:Nucleotide transport and metabolism; |
| N840_1977 | fliG | 0.274 | COG1536 | N:Cell motility |
| N840_1979 | fliI | 0.276 | COG1157 | N:Cell motility;U:Intracellular trafficking, secretion, and vesicular transport |
| N840_2154 | yehA | 0.165 | ENOG410XTMS | S:Function unknown |
| N840_1089 | flgJ | 0.291 | COG1705 | N:Cell motility;U:Intracellular trafficking, secretion, and vesicular transport |
| N840_4387 | yjhP | 0.488 | ENOG410XQ9J | S:Function unknown |
| N840_2270 | atoA | 0.078 | COG2057 | I:Lipid transport and metabolism |
| N840_2690 | yfjP | 0.415 | COG3596 | S:Function unknown |
| N840_2615 | mltF | 0.498 | COG4623 | M:Cell wall/membrane/envelope biogenesis |
| N840_1841 | yeaX | 0.433 | COG1018 | P:Inorganic ion transport and metabolism |
| N840_2397 | intS | 0.392 | COG0582 | M:Cell wall/membrane/envelope biogenesis |
| N840_1831 | yoaK | 0.37 | ENOG410Y5TS | S:Function unknown |
| N840_1182 | ymgG | 0.416 | ------ | ------ |
| N840_0297 | ykgJ | 0.325 | COG0727 | S:Function unknown |
| N840_3662 | yibW | 0.042 | COG3209 | M:Cell wall/membrane/envelope biogenesis |
| N840_1705 | ydhX | 0.379 | COG0437 | C:Energy production and conversion |
| N840_1559 | marB | 0.155 | ENOG410Y64J | P:Inorganic ion transport and metabolism |
| N840_2285 | yfaH | 0.028 | ENOG410XP61 | T:Signal transduction mechanisms |
| N840_0337 | yahM | 0.39 | ------ | ------ |
| N840_2191 | yeiS | 0.305 | ENOG41129X4 | S:Function unknown |
| N840_1943 | azuC | 0.456 | ENOG410Y5WR | S:Function unknown |
| N840_1932 | motA | 0.347 | COG1291 | N:Cell motility |
| N840_3534 | nikR | 0.471 | COG0864 | K:Transcription |
| N840_0617 | ybdM | 0.486 | COG1475 | K:Transcription |
| N840_0328 | yahC | 0.463 | ENOG41120CM | S:Function unknown |
| N840_1293 | ymiA | 0.434 | ENOG4112AAA | S:Function unknown |
| N840_3839 | ilvX | 0.317 | ------ | ------ |
| N840_2687 | yfjM | 0.259 | ENOG410ZDU4 | S:Function unknown |
| N840_2593 | iroK | 0.435 | ------ | ------ |
| N840_3762 | cbrA | 0.408 | COG0644 | S:Function unknown |
| N840_1924 | cheY | 0.459 | COG0784 | T:Signal transduction mechanisms |
| N840_3011 | yggM | 0.355 | ENOG410Y9EZ | S:Function unknown |
| N840_3629 | xylG | 0.454 | COG1129 | G:Carbohydrate transport and metabolism |
| N840_1647 | uidC | 0.417 | ENOG410XU8G | U:Intracellular trafficking, secretion, and vesicular transport |
| N840_2324 | yfbP | 0.447 | ENOG410Y787 | S:Function unknown |
| N840_1227 | ldrA | 0.437 | ------ | ------ |
| N840_1778 | ves | 0.38 | COG3758 | S:Function unknown |
| N840_3758 | ibpB | 0.376 | COG0071 | O:Posttranslational modification, protein turnover, chaperones |
| N840_0253 | yafY | 0.491 | COG2378 | K:Transcription |
| N840_3377 | gspC | 0.361 | COG3031 | U:Intracellular trafficking, secretion, and vesicular transport |
| N840_4331 | yjgL | 0.423 | ------ | ------ |
| N840_0700 | ybfP | 0.414 | ------ | ------ |
| N840_3429 | yhfS | 0.472 | ENOG410Z1QN | E:Amino acid transport and metabolism |
| N840_0349 | cynS | 0.454 | COG1513 | P:Inorganic ion transport and metabolism |
| N840_1183 | ymgI | 0.302 | ------ | ------ |
| N840_0952 | ycbV | 0.27 | COG3539 | N:Cell motility;U:Intracellular trafficking, secretion, and vesicular transport |
| N840_1147 | ymfE | 0.453 | ENOG410Y9BP | S:Function unknown |
| N840_1231 | ldrC | 0.431 | ------ | ------ |
| N840_4147 | nrfC | 0.351 | COG0437 | C:Energy production and conversion |
| N840_2253 | napD | 0.349 | COG3062 | P:Inorganic ion transport and metabolism |
| N840_0578 | envY | 0.461 | ENOG410Y9V9 | K:Transcription; |
| N840_1978 | fliH | 0.303 | COG1317 | N:Cell motility |
| N840_2940 | ygfS | 0.328 | COG1142 | S:Function unknown |
| N840_1922 | flhB | 0.271 | COG1377 | N:Cell motility |
| N840_0267 | afuB | 0.218 | COG1178 | E:Amino acid transport and metabolism |
| N840_4146 | nrfB | 0.477 | ENOG411128Q | C:Energy production and conversion |
| N840_3962 | yiiF | 0.462 | ENOG410XWG0 | S:Function unknown |
| N840_3092 | ygiL | 0.164 | ENOG4111SNU | S:Function unknown |
| N840_0505 | ylbH | 0.048 | COG3209 | M:Cell wall/membrane/envelope biogenesis |
| N840_3596 | rdlD | 0.485 | ------ | ------ |
| N840_4111 | yjbI | 0.315 | COG1357 | S:Function unknown |
| N840_4223 | yjeJ | 0.417 | ENOG4111VQX | S:Function unknown |
| N840_1523 | yddB | 0.448 | ENOG410XSVA | P:Inorganic ion transport and metabolism |
| N840_2427 | ypdI | 0.097 | ------ | ------ |
| N840_3000 | endA | 0.433 | COG2356 | L:Replication, recombination and repair |
| N840_4426 | mcrC | 0.483 | COG4268 | V:Defense mechanisms |
| N840_1530 | ydeQ | 0.174 | ENOG410YESZ | S:Function unknown |
| N840_4391 | yjhR | 0.44 | COG1112 | L:Replication, recombination and repair |
| N840_4285 | yjfZ | 0.407 | ------ | ------ |
| N840_3113 | ttdR | 0.431 | ENOG410XNQA | S:Function unknown |
| N840_1821 | yeaI | 0.479 | COG2199 | S:Function unknown |
| N840_0504 | ybbC | 0.324 | ------ | ------ |
| N840_2719 | ygaV | 0.365 | COG0640 | K:Transcription |
| N840_1379 | sieB | 0.059 | ------ | ------ |
| N840_0660 | djlB | 0.216 | ENOG410XVEN; | O:Posttranslational modification, protein turnover, chaperones; |
| N840_2500 | yffQ | 0.419 | ------ | ------ |
| N840_1156 | ymfM | 0.44 | ------ | ------ |
| N840_2115 | yegK | 0.343 | ENOG4111YVJ | S:Function unknown |
| N840_2765 | hydN | 0.433 | COG1142 | O:Posttranslational modification, protein turnover, chaperones |
| N840_0350 | cynX | 0.478 | COG2807 | G:Carbohydrate transport and metabolism |
| N840_4429 | symR | 0.458 | ------ | ------ |
| N840_1984 | fliN | 0.357 | COG1886 | N:Cell motility |
| N840_1986 | fliP | 0.176 | COG1338 | N:Cell motility |
| N840_3841 | ilvM | 0.463 | COG3978 | E:Amino acid transport and metabolism |
| N840_3046 | hybF | 0.476 | COG0375 | E:Amino acid transport and metabolism |
| N840_1944 | yecR | 0.306 | ENOG41121GA | S:Function unknown |
| N840_2716 | csiR | 0.479 | COG1802 | Q:Secondary metabolites biosynthesis, transport and catabolism |
| N840_3660 | yibG | 0.122 | ------ | ------ |
| N840_1445 | rydC | 0.497 | ------ | ------ |
| N840_0307 | ykgR | 0.222 | ------ | ------ |
| N840_2710 | ygaQ | 0.162 | COG0366 | L:Replication, recombination and repair |
| N840_4148 | nrfD | 0.495 | COG3301 | P:Inorganic ion transport and metabolism |
| N840_1965 | fliS | 0.384 | COG1516 | N:Cell motility |
| N840_1525 | ydeM | 0.319 | COG0641 | S:Function unknown |
| N840_3978 | rhaR | 0.495 | ENOG410XYX0 | K:Transcription |
| N840_1146 | ymfD | 0.498 | ENOG410YCU8 | S:Function unknown |
| N840_1925 | cheB | 0.477 | COG2201 | T:Signal transduction mechanisms |
| N840_0999 | insB1 | 0.18 | COG1662 | L:Replication, recombination and repair |
| N840_1751 | pheM | 0.496 | ------ | ------ |

**Table S2. The information of up-regulated genes in *E.coli* C321.ΔA**

| Gene_ID | Gene name | Fold Change | COG ID | COG_Functional Categories |
| --- | --- | --- | --- | --- |
| N840_2262 | ompC | 20.466 | COG3203 | H:Coenzyme transport and metabolism |
| N840_1521 | gadB | 132.967 | COG0076 | E:Amino acid transport and metabolism |
| N840_1520 | gadC | 159.205 | COG0531 | S:Function unknown |
| N840_3563 | hdeB | 235.757 | ENOG41125MZ | S:Function unknown |
| N840_2039 | flu | 24.783 | COG3468 | L:Replication, recombination and repair |
| N840_1321 | puuC | 37.429 | COG1012 | K:Transcription |
| N840_1715 | sufD | 13.825 | COG0719 | O:Posttranslational modification, protein turnover, chaperones |
| N840_3126 | patA | 14.637 | COG4992 | K:Transcription |
| N840_1898 | znuA | 7.33 | COG4531 | P:Inorganic ion transport and metabolism |
| N840_4496 | gam | 18.958 | ENOG410XWXJ | S:Function unknown |
| N840_1717 | sufB | 9.802 | COG0719 | O:Posttranslational modification, protein turnover, chaperones |
| N840_0491 | glsA | 65.914 | COG2066 | E:Amino acid transport and metabolism |
| N840_1323 | puuE | 37.758 | COG0160 | E:Amino acid transport and metabolism |
| N840_4490 | bet | 19.139 | ENOG4111HT0 | L:Replication, recombination and repair |
| N840_1716 | sufC | 16.464 | COG0396 | O:Posttranslational modification, protein turnover, chaperones |
| N840_1322 | puuB | 76.047 | ENOG410XWBT | C:Energy production and conversion |
| N840_1318 | puuA | 13.106 | COG0174 | E:Amino acid transport and metabolism |
| N840_3564 | hdeA | 350.565 | ENOG41128BT | O:Posttranslational modification, protein turnover, chaperones |
| N840_4495 | ea22 | 18.835 | ENOG410Y7W8 | S:Function unknown; |
| N840_4497 | N | 8.865 | ENOG410YH6K | S:Function unknown |
| N840_4488 | int | 8.745 | COG0582 | G:Carbohydrate transport and metabolism |
| N840_0492 | ybaT | 24.009 | COG0531 | E:Amino acid transport and metabolism |
| N840_0779 | ybhD | 9.846 | ENOG410XRIM | K:Transcription |
| N840_4498 | ea10 | 33.087 | ------ | ------ |
| N840_4492 | exo | 30.032 | ENOG4111FJH | L:Replication, recombination and repair |
| N840_1803 | ansA | 9.962 | COG0252 | E:Amino acid transport and metabolism |
| N840_0783 | ybhC | 5.923 | COG4677 | G:Carbohydrate transport and metabolism |
| N840_4268 | aidB | 10.39 | COG1960 | S:Function unknown |
| N840_3565 | hdeD | 820.377 | COG3247 | S:Function unknown |
| N840_1714 | sufS | 8.01 | COG0520 | E:Amino acid transport and metabolism |
| N840_0777 | ybhA | 6.054 | COG0561 | P:Inorganic ion transport and metabolism |
| N840_4501 | ral | 13.229 | ------ | ------ |
| N840_2010 | zinT | 19.369 | COG3443 | S:Function unknown |
| N840_1497 | narZ | 10.948 | COG5013 | S:Function unknown |
| N840_2181 | yohC | 5.013 | ENOG4111H2T | S:Function unknown |
| N840_3567 | gadE | 358.382 | ENOG410YREC | K:Transcription |
| N840_4102 | yjbT | 72.759 | ------ | ------ |
| N840_3936 | csrC | 9.893 | ------ | ------ |
| N840_3571 | gadY | 56.408 | ------ | ------ |
| N840_3570 | gadW | 17.888 | ENOG41101VY | K:Transcription; |
| N840_3900 | metE | 12.984 | COG0620 | E:Amino acid transport and metabolism |
| N840_1899 | znuC | 4.533 | COG1121 | P:Inorganic ion transport and metabolism |
| N840_1496 | narY | 9.528 | COG1140 | S:Function unknown |
| N840_4332 | argI | 18.324 | COG0078 | K:Transcription |
| N840_3560 | slp | 116.584 | COG3065 | L:Replication, recombination and repair |
| N840_1712 | ynhG | 4.181 | COG1376 | M:Cell wall/membrane/envelope biogenesis |
| N840_2959 | gcvH | 4.014 | COG0509 | E:Amino acid transport and metabolism |
| N840_1317 | puuP | 8.67 | COG0531 | S:Function unknown |
| N840_1012 | cbpA | 5.286 | COG2214 | O:Posttranslational modification, protein turnover, chaperones |
| N840_3980 | sodA | 6.055 | COG0605 | K:Transcription |
| N840_1498 | narU | 12.982 | COG2223 | S:Function unknown |
| N840_1017 | ymdF | 7.103 | COG3729 | S:Function unknown |
| N840_3259 | yrbL | 3.037 | ENOG410ZFFS | S:Function unknown |
| N840_4420 | mdtM | 3.921 | ENOG410XT98 | S:Function unknown |
| N840_1718 | sufA | 5.639 | COG0316 | O:Posttranslational modification, protein turnover, chaperones |
| N840_3572 | gadX | 10.663 | ENOG410XRJG | K:Transcription |
| N840_3742 | ilvB | 3.3 | COG0028 | F:Nucleotide transport and metabolism |
| N840_1897 | yebA | 3.747 | COG0739 | M:Cell wall/membrane/envelope biogenesis |
| N840_0767 | aroG | 3.548 | COG0722 | E:Amino acid transport and metabolism |
| N840_1049 | csgD | 161.58 | COG2771 | U:Intracellular trafficking, secretion, and vesicular transport |
| N840_4505 | cIII | 25.2 | ------ | ------ |
| N840_3569 | mdtF | 71.163 | COG0841 | K:Transcription |
| N840_3568 | mdtE | 227.534 | ENOG410XNVN | K:Transcription |
| N840_0981 | hyaA | 5.155 | COG1740 | C:Energy production and conversion |
| N840_4449 | fhuF | 8.847 | COG4114 | S:Function unknown |
| N840_0850 | mdfA | 9.07 | ENOG410XT98 | G:Carbohydrate transport and metabolism |
| N840_4500 | xis | 10.211 | ------ | ------ |
| N840_1047 | csgF | 142.436 | ENOG4111JSD | U:Intracellular trafficking, secretion, and vesicular transport |
| N840_2631 | rseA | 3.12 | COG3073 | T:Signal transduction mechanisms |
| N840_3059 | exbB | 6.15 | COG0811 | S:Function unknown |
| N840_1217 | ychH | 6.327 | ENOG4111VC3 | S:Function unknown |
| N840_2958 | gcvP | 3.236 | COG1003 | E:Amino acid transport and metabolism |
| N840_1820 | yeaH | 3.708 | COG2718 | S:Function unknown |
| N840_0279 | argF | 6.713 | COG0078 | E:Amino acid transport and metabolism |
| N840_0982 | hyaB | 3.828 | COG0374 | C:Energy production and conversion |
| N840_1695 | cfa | 3.647 | COG2230 | M:Cell wall/membrane/envelope biogenesis |
| N840_2421 | evgS | 8.21 | COG0784 | T:Signal transduction mechanisms |
| N840_0769 | galT | 2.959 | COG1085 | G:Carbohydrate transport and metabolism |
| N840_3573 | gadA | 443.134 | COG0076 | K:Transcription |
| N840_4132 | uvrA | 2.776 | COG0178 | L:Replication, recombination and repair |
| N840_1048 | csgE | 175.499 | ENOG4111JH9 | U:Intracellular trafficking, secretion, and vesicular transport |
| N840_1011 | cbpM | 4.178 | ENOG411205W | S:Function unknown |
| N840_3894 | rhtC | 13.223 | COG1280 | L:Replication, recombination and repair |
| N840_0390 | psiF | 3.577 | ENOG41121V4 | S:Function unknown |
| N840_0869 | artJ | 3.333 | COG0834 | J:Translation, ribosomal structure and biogenesis |
| N840_3462 | feoB | 5.366 | COG0370 | P:Inorganic ion transport and metabolism |
| N840_3461 | feoA | 6.179 | COG1918 | P:Inorganic ion transport and metabolism |
| N840_1319 | puuD | 12.947 | COG2071 | S:Function unknown |
| N840_4368 | fecA | 5.096 | COG4772 | P:Inorganic ion transport and metabolism |
| N840_0824 | mntS | 5.023 | ENOG410Y3FH | S:Function unknown |
| N840_2960 | gcvT | 3.342 | COG0404 | E:Amino acid transport and metabolism |
| N840_1051 | csgA | 304.118 | ENOG4111QFZ | G:Carbohydrate transport and metabolism |
| N840_1720 | ydiH | 4.21 | ENOG4112CA3 | S:Function unknown |
| N840_1046 | csgG | 46.528 | COG1462 | M:Cell wall/membrane/envelope biogenesis |
| N840_0822 | ompX | 3.005 | COG3637 | M:Cell wall/membrane/envelope biogenesis |
| N840_1713 | sufE | 6.781 | COG2166 | S:Function unknown |
| N840_2727 | nrdE | 4.713 | COG0209 | F:Nucleotide transport and metabolism |
| N840_1320 | puuR | 4.656 | COG1396 | K:Transcription; |
| N840_0392 | proC | 2.668 | COG0345 | E:Amino acid transport and metabolism |
| N840_1782 | astD | 3.328 | COG1012 | E:Amino acid transport and metabolism |
| N840_4299 | ytfL | 3.666 | COG1253 | P:Inorganic ion transport and metabolism |
| N840_3415 | fic | 3.178 | COG2184 | D:Cell cycle control, cell division, chromosome partitioning |
| N840_3575 | treF | 4.445 | COG1626 | P:Inorganic ion transport and metabolism |
| N840_2725 | nrdH | 5.223 | COG0695 | S:Function unknown |
| N840_1916 | cutC | 2.969 | COG3142 | P:Inorganic ion transport and metabolism |
| N840_4317 | nrdD | 3.08 | COG1328 | F:Nucleotide transport and metabolism |
| N840_1050 | csgB | 1532.928 | ENOG4111G8B | M:Cell wall/membrane/envelope biogenesis |
| N840_2026 | erfK | 3.587 | COG1376 | K:Transcription |
| N840_3224 | argG | 2.535 | COG0137 | E:Amino acid transport and metabolism |
| N840_1991 | yodD | 4.125 | ENOG411261I | S:Function unknown |
| N840_4182 | yjdN | 3.47 | COG2764 | S:Function unknown |
| N840_1781 | astB | 3.014 | COG3724 | E:Amino acid transport and metabolism |
| N840_1804 | pncA | 4.568 | COG1335 | E:Amino acid transport and metabolism |
| N840_1495 | narW | 10.628 | COG2180 | S:Function unknown |
| N840_3203 | yhbO | 4.674 | COG0693 | S:Function unknown |
| N840_2057 | yeeY | 2.582 | ENOG410XP36 | K:Transcription |
| N840_1900 | znuB | 4.378 | COG1108 | P:Inorganic ion transport and metabolism |
| N840_0121 | gcd | 4.47 | COG4993 | G:Carbohydrate transport and metabolism |
| N840_4030 | argH | 2.755 | COG0165 | E:Amino acid transport and metabolism |
| N840_2038 | isrC | 79.481 | COG3468 | L:Replication, recombination and repair |
| N840_0771 | modF | 2.973 | COG1119 | P:Inorganic ion transport and metabolism |
| N840_2873 | argA | 3.158 | COG0548 | E:Amino acid transport and metabolism |
| N840_2133 | yegS | 2.955 | COG1597 | I:Lipid transport and metabolism |
| N840_1967 | amyA | 2.499 | COG0366 | K:Transcription |
| N840_1784 | astC | 3.11 | COG4992 | E:Amino acid transport and metabolism |
| N840_1993 | yedP | 2.733 | COG3769 | S:Function unknown |
| N840_4465 | deoC | 2.445 | COG0274 | F:Nucleotide transport and metabolism |
| N840_0765 | zitB | 2.714 | COG1230 | P:Inorganic ion transport and metabolism |
| N840_0495 | qmcA | 2.213 | COG0330 | O:Posttranslational modification, protein turnover, chaperones |
| N840_1398 | pinR | 38.024 | COG1961 | S:Function unknown |
| N840_3058 | exbD | 3.653 | COG0848 | S:Function unknown |
| N840_4116 | lexA | 2.417 | COG1974 | M:Cell wall/membrane/envelope biogenesis |
| N840_3562 | yhiD | 90.676 | COG1285 | S:Function unknown |
| N840_0608 | entC | 10.31 | COG1169 | H:Coenzyme transport and metabolism |
| N840_0032 | carB | 3.589 | COG0458 | F:Nucleotide transport and metabolism |
| N840_0147 | fhuA | 3.767 | COG1629 | P:Inorganic ion transport and metabolism |
| N840_2632 | rpoE | 2.346 | COG1595 | K:Transcription |
| N840_1937 | otsB | 2.414 | COG1877 | G:Carbohydrate transport and metabolism |
| N840_0316 | ykgC | 3.948 | COG1249 | S:Function unknown |
| N840_1783 | astA | 2.869 | COG3138 | E:Amino acid transport and metabolism |
| N840_1200 | ycgB | 2.416 | COG2719 | S:Function unknown |
| N840_0031 | carA | 4.893 | COG0505 | F:Nucleotide transport and metabolism |
| N840_2127 | yegP | 2.484 | COG3422 | S:Function unknown |
| N840_2846 | csrB | 2.714 | ------ | ------ |
| N840_2183 | yohF | 2.813 | COG1028 | S:Function unknown |
| N840_3265 | gltB | 2.36 | COG0070 | E:Amino acid transport and metabolism |
| N840_2613 | glmY | 5.865 | ------ | ------ |
| N840_1780 | astE | 2.794 | COG2988 | E:Amino acid transport and metabolism |
| N840_3578 | yhjD | 3.537 | COG1295 | S:Function unknown |
| N840_3390 | bfd | 8.351 | COG2906 | S:Function unknown |
| N840_0605 | fepD | 10.337 | COG0609 | P:Inorganic ion transport and metabolism |
| N840_0175 | skp | 2.099 | COG2825 | M:Cell wall/membrane/envelope biogenesis |
| N840_3652 | aldB | 3.07 | COG1012 | S:Function unknown |
| N840_0880 | poxB | 2.455 | COG0028 | E:Amino acid transport and metabolism |
| N840_0745 | cydA | 2.618 | COG1271 | C:Energy production and conversion |
| N840_1494 | narV | 7.827 | COG2181 | S:Function unknown |
| N840_3266 | gltD | 2.741 | COG0493 | E:Amino acid transport and metabolism |
| N840_0973 | yccT | 3.474 | COG3110 | S:Function unknown |
| N840_2040 | yeeR | 4.068 | ENOG410XXEN | S:Function unknown |
| N840_1875 | yebV | 3.735 | ENOG411247I | S:Function unknown |
| N840_0721 | phr | 2.139 | COG0415 | L:Replication, recombination and repair |
| N840_0058 | polB | 2.351 | COG0417 | L:Replication, recombination and repair |
| N840_1889 | yebG | 2.368 | COG3141 | S:Function unknown |
| N840_2443 | mntH | 4.276 | COG1914 | P:Inorganic ion transport and metabolism |
| N840_2529 | ypfJ | 2.208 | COG2321 | S:Function unknown |
| N840_0152 | clcA | 2.248 | COG0038 | P:Inorganic ion transport and metabolism |
| N840_1844 | yeaY | 2.73 | COG3065 | M:Cell wall/membrane/envelope biogenesis |
| N840_0720 | ybgA | 2.362 | COG3272 | S:Function unknown |
| N840_1469 | ydcS | 2.918 | ENOG410XUAZ | G:Carbohydrate transport and metabolism |
| N840_4028 | argC | 3.084 | COG0002 | E:Amino acid transport and metabolism |
| N840_0609 | entE | 7.665 | COG1021 | Q:Secondary metabolites biosynthesis, transport and catabolism |
| N840_1484 | yncG | 3.688 | COG0625 | S:Function unknown |
| N840_0766 | ybgS | 2.601 | ENOG4111NF4 | S:Function unknown |
| N840_3544 | yhiM | 210.801 | ENOG410ZE9J | S:Function unknown |
| N840_3501 | yhhA | 2.209 | ENOG411273Y | S:Function unknown |
| N840_3566 | arrS | 188.04 | ------ | ------ |
| N840_2185 | yohP | 4.493 | ------ | ------ |
| N840_2288 | glpA | 4.874 | COG0578 | C:Energy production and conversion |
| N840_4295 | cysQ | 2.761 | COG1218 | P:Inorganic ion transport and metabolism |
| N840_1992 | dsrA | 2.634 | ------ | ------ |
| N840_4229 | blc | 2.281 | COG3040 | M:Cell wall/membrane/envelope biogenesis |
| N840_0606 | entS | 7.394 | COG0477 | P:Inorganic ion transport and metabolism |
| N840_2970 | serA | 2.126 | COG0111 | H:Coenzyme transport and metabolism |
| N840_1286 | rluB | 2.136 | COG1187 | S:Function unknown |
| N840_0808 | ybiB | 2.031 | COG0547 | E:Amino acid transport and metabolism |
| N840_1643 | fumC | 2.184 | COG0114 | C:Energy production and conversion |
| N840_1588 | cspB | 3.344 | COG1278 | S:Function unknown |
| N840_0676 | ubiF | 2.201 | COG0654 | J:Translation, ribosomal structure and biogenesis |
| N840_1276 | yciG | 3.744 | COG3729 | S:Function unknown |
| N840_2630 | rseB | 2.158 | COG3026 | T:Signal transduction mechanisms |
| N840_3580 | yhjG | 3.182 | COG2982 | S:Function unknown |
| N840_3416 | yhfG | 3.805 | ENOG4112AAY | S:Function unknown |
| N840_0805 | rhlE | 2.285 | COG0513 | L:Replication, recombination and repair |
| N840_2281 | nrdA | 2.185 | COG0209 | F:Nucleotide transport and metabolism |
| N840_2957 | ygfF | 2.446 | ENOG410XNUN | S:Function unknown |
| N840_3714 | dinD | 2.378 | ENOG410XQQP | S:Function unknown |
| N840_0306 | ykgM | 8.897 | COG0254 | J:Translation, ribosomal structure and biogenesis |
| N840_1001 | cspG | 4.02 | COG1278 | K:Transcription |
| N840_1688 | ydhO | 3.01 | COG0791 | O:Posttranslational modification, protein turnover, chaperones |
| N840_0637 | pagP | 5.445 | ENOG410XTHE | M:Cell wall/membrane/envelope biogenesis |
| N840_1877 | pphA | 2.661 | COG0639 | S:Function unknown |
| N840_4506 | kil | 43.403 | ------ | ------ |
| N840_0598 | fepA | 2.941 | COG4771 | P:Inorganic ion transport and metabolism |
| N840_3945 | yihM | 7.327 | ENOG410ZVGB | S:Function unknown |
| N840_0807 | dinG | 2.118 | COG1199 | L:Replication, recombination and repair |
| N840_2463 | ligA | 2.125 | COG0272 | L:Replication, recombination and repair |
| N840_3632 | bax | 2.502 | COG2992 | S:Function unknown |
| N840_0335 | yahK | 2.175 | COG1064 | C:Energy production and conversion |
| N840_0772 | modE | 2.26 | COG2005 | K:Transcription |
| N840_3402 | slyD | 2.087 | COG1047 | S:Function unknown |
| N840_4029 | argB | 2.594 | COG0548 | E:Amino acid transport and metabolism |
| N840_0593 | ybdK | 2.249 | COG2170 | S:Function unknown |
| N840_0712 | ybfA | 2.507 | ENOG41123Q8 | S:Function unknown |
| N840_4056 | rplL | 2.028 | COG0222 | J:Translation, ribosomal structure and biogenesis |
| N840_2172 | mlrA | 2.27 | COG0789 | T:Signal transduction mechanisms |
| N840_1472 | ydcV | 3.303 | ENOG410XPIA | P:Inorganic ion transport and metabolism |
| N840_1452 | ydcJ | 2.56 | COG5383 | S:Function unknown |
| N840_3613 | yiaG | 3.135 | COG2944 | K:Transcription |
| N840_1433 | ydbC | 2.867 | ENOG410XRV3 | C:Energy production and conversion |
| N840_1473 | patD | 3.279 | COG1012 | P:Inorganic ion transport and metabolism |
| N840_1702 | ydhS | 2.128 | COG4529 | S:Function unknown |
| N840_3998 | glpF | 2.994 | COG0580 | C:Energy production and conversion |
| N840_0713 | rhsC | 2.643 | COG3209 | S:Function unknown |
| N840_2829 | queE | 3.228 | COG0602 | O:Posttranslational modification, protein turnover, chaperones |
| N840_3606 | yhjY | 2.225 | COG5571 | N:Cell motility |
| N840_2215 | fruB | 2.591 | COG1925 | Q:Secondary metabolites biosynthesis, transport and catabolism |
| N840_1054 | ymdB | 2.103 | COG2110 | S:Function unknown |
| N840_2341 | yfbS | 2.143 | COG0471 | S:Function unknown |
| N840_0458 | ybaY | 2.216 | COG3126 | S:Function unknown |
| N840_4460 | ytjA | 3.696 | ENOG410Z433 | S:Function unknown |
| N840_2562 | guaB | 2.165 | COG0516 | F:Nucleotide transport and metabolism; |
| N840_2388 | sixA | 2.126 | COG2062 | T:Signal transduction mechanisms |
| N840_0395 | yaiA | 2.119 | ENOG41121PA | S:Function unknown |
| N840_4370 | fecI | 5.84 | COG1595 | T:Signal transduction mechanisms |
| N840_1470 | ydcT | 2.495 | COG3842 | G:Carbohydrate transport and metabolism |
| N840_1627 | ynfM | 2.609 | ENOG410XP8J | G:Carbohydrate transport and metabolism |
| N840_2284 | inaA | 2.006 | ENOG4111Q68 | T:Signal transduction mechanisms |
| N840_0448 | fadM | 3.037 | COG0824 | S:Function unknown |
| N840_0607 | fepB | 3.686 | COG4592 | P:Inorganic ion transport and metabolism |
| N840_4347 | yjgB | 2.033 | COG1064 | K:Transcription |
| N840_3901 | ysgA | 2.296 | COG0412 | E:Amino acid transport and metabolism |
| N840_3547 | uspB | 2.559 | ENOG4111J5S | S:Function unknown |
| N840_1061 | mdtG | 2.217 | ENOG410ZVCH | G:Carbohydrate transport and metabolism |
| N840_2214 | fruK | 2.079 | COG1105 | Q:Secondary metabolites biosynthesis, transport and catabolism |
| N840_1269 | tonB | 2.458 | COG0810 | M:Cell wall/membrane/envelope biogenesis |
| N840_3732 | yicS | 3.207 | ENOG4111WZH | S:Function unknown |
| N840_1455 | rimL | 2.565 | COG1670 | S:Function unknown |
| N840_1471 | ydcU | 2.558 | COG1176 | G:Carbohydrate transport and metabolism |
| N840_0296 | yagU | 4.874 | COG3477 | S:Function unknown |
| N840_3612 | yiaF | 2.478 | ENOG410YECY | S:Function unknown |
| N840_0763 | nadA | 2.056 | COG0379 | H:Coenzyme transport and metabolism |
| N840_0624 | ybdR | 2.034 | COG1063 | E:Amino acid transport and metabolism |
| N840_2726 | nrdI | 4.778 | COG1780 | F:Nucleotide transport and metabolism |
| N840_1719 | rydB | 4.125 | ------ | ------ |
| N840_2653 | yfiH | 2.023 | COG1496 | S:Function unknown |
| N840_2629 | rseC | 2.285 | COG3086 | T:Signal transduction mechanisms |
| N840_3561 | dctR | 71.227 | COG2771 | L:Replication, recombination and repair |
| N840_4270 | bsmA | 2.387 | ENOG41123U1 | S:Function unknown |
| N840_0734 | sdhC | 2.256 | COG2009 | C:Energy production and conversion |
| N840_3291 | yhcN | 2.438 | ENOG4111Z93 | S:Function unknown |
| N840_4459 | osmY | 2.167 | ENOG410YCX0 | S:Function unknown; |
| N840_0391 | yaiC | 4.501 | ENOG410XNMH | T:Signal transduction mechanisms |
| N840_0220 | ivy | 2.817 | ENOG4111K9M | S:Function unknown |
| N840_1029 | efeO | 3.282 | COG2822 | P:Inorganic ion transport and metabolism |
| N840_0813 | fiu | 3.364 | COG4774 | P:Inorganic ion transport and metabolism |
| N840_3741 | ilvN | 2.661 | COG0440 | F:Nucleotide transport and metabolism |
| N840_0295 | paoA | 2.425 | COG2080 | C:Energy production and conversion |
| N840_2258 | yojI | 2.393 | COG4615 | V:Defense mechanisms |
| N840_0797 | clsB | 2.182 | COG1502 | I:Lipid transport and metabolism |
| N840_3614 | cspA | 2.613 | COG1278 | K:Transcription |
| N840_4499 | ea8.5 | 2.008 | ------ | ------ |
| N840_1956 | sdiA | 2.017 | COG2771 | S:Function unknown |
| N840_1353 | ynaI | 2.07 | COG0668 | K:Transcription |
| N840_2817 | cysJ | 2.009 | COG0369 | P:Inorganic ion transport and metabolism |
| N840_3463 | feoC | 3.375 | ENOG411247E | K:Transcription |
| N840_2350 | yfcF | 2.022 | COG0625 | S:Function unknown |
| N840_0984 | hyaD | 2.316 | COG0680 | C:Energy production and conversion |
| N840_2844 | truC | 2.014 | COG0564 | G:Carbohydrate transport and metabolism |
| N840_3734 | yicN | 4.018 | ENOG4111H7S | S:Function unknown |
| N840_0561 | insH1 | 4873.69 | COG3039 | L:Replication, recombination and repair |
| N840_1867 | yebQ | 2.115 | ENOG410XNN3 | P:Inorganic ion transport and metabolism |
| N840_2944 | idi | 2.229 | COG1443 | I:Lipid transport and metabolism |
| N840_3944 | yihL | 2.904 | COG2188 | T:Signal transduction mechanisms |
| N840_1002 | ymcE | 3.445 | ------ | ------ |
| N840_2289 | glpB | 2.939 | COG3075 | E:Amino acid transport and metabolism |
| N840_0983 | hyaC | 2.554 | COG1969 | C:Energy production and conversion |
| N840_2031 | insH1 | 2.105 | COG3039 | H:Coenzyme transport and metabolism |
| N840_2728 | nrdF | 3.022 | COG0208 | F:Nucleotide transport and metabolism |
| N840_1207 | ymgE | 2.403 | COG2261 | S:Function unknown |
| N840_0798 | ybhP | 2 | COG3568 | S:Function unknown |
| N840_2261 | apbE | 2.169 | COG1477 | H:Coenzyme transport and metabolism |
| N840_0388 | iraP | 2.21 | ENOG4111YMR | T:Signal transduction mechanisms |
| N840_3551 | prlC | 2.244 | COG0339 | M:Cell wall/membrane/envelope biogenesis |
| N840_1480 | mcbR | 2.146 | COG1802 | K:Transcription |
| N840_3019 | nupG | 2.071 | ENOG410ZVFU | P:Inorganic ion transport and metabolism |
| N840_0710 | kdpA | 5.509 | COG2060 | P:Inorganic ion transport and metabolism |
| N840_3997 | glpK | 2.036 | COG0554 | C:Energy production and conversion |
| N840_3545 | yhiN | 2.201 | COG2081 | S:Function unknown |
| N840_2351 | yfcG | 2.096 | COG0625 | S:Function unknown |
| N840_2290 | glpC | 2.682 | COG0247 | E:Amino acid transport and metabolism |
| N840_3543 | yhiL | 3.599 | ------ | ------ |
| N840_1474 | yncL | 2.112 | ------ | ------ |
| N840_0189 | arfB | 2.017 | COG1186 | J:Translation, ribosomal structure and biogenesis |
| N840_4138 | ryjA | 2.135 | ------ | ------ |
| N840_1658 | cnu | 3.874 | ENOG4111UG9 | S:Function unknown |
| N840_2430 | ypdK | 2.435 | ------ | ------ |
| N840_0833 | fsaA | 2.142 | COG0176 | O:Posttranslational modification, protein turnover, chaperones |
| N840_4227 | ecnB | 2.154 | ENOG410Y4IR | S:Function unknown |
| N840_2170 | yehT | 2.777 | COG3279 | T:Signal transduction mechanisms |
| N840_0294 | paoB | 2.034 | COG1319 | C:Energy production and conversion |
| N840_4367 | fecB | 2.101 | COG4594 | P:Inorganic ion transport and metabolism |
| N840_3559 | insH1 | 2.597 | COG3039 | L:Replication, recombination and repair |
| N840_1573 | ydfK | 4.565 | ENOG411246H | K:Transcription |
| N840_3946 | yihN | 7.99 | ENOG410XQHZ | G:Carbohydrate transport and metabolism |
| N840_3579 | yhjE | 2.972 | ENOG410XP7I | S:Function unknown |
| N840_2722 | alaE | 2.322 | ENOG4111J5P | U:Intracellular trafficking, secretion, and vesicular transport |
| N840_0985 | hyaE | 2.253 | ENOG4111JYZ | K:Transcription |
| N840_0781 | ybhI | 10.295 | COG0471 | S:Function unknown |
| N840_0610 | entB | 4.401 | COG3433 | Q:Secondary metabolites biosynthesis, transport and catabolism |
| N840_4413 | yjiH | 2.376 | COG3314 | S:Function unknown |
| N840_3846 | ilvC | 2.274 | COG0059 | E:Amino acid transport and metabolism |
| N840_1350 | ycjY | 2.015 | COG1073 | E:Amino acid transport and metabolism |
| N840_0473 | ybaN | 2.545 | COG2832 | S:Function unknown |
| N840_2263 | micF | 8.968 | ------ | ------ |
| N840_0544 | exoD | 8.053 | ENOG4111FJH | L:Replication, recombination and repair |
| N840_2401 | tfaS | 2.091 | ENOG4111IKE | S:Function unknown |
| N840_0078 | ilvH | 2.391 | COG0440 | E:Amino acid transport and metabolism |
| N840_3169 | tdcA | 3.113 | ENOG410XW1S | K:Transcription |
| N840_3177 | garP | 4.49 | ENOG410XPWC | S:Function unknown |
| N840_3550 | rsmJ | 2.111 | ENOG410XPRJ | M:Cell wall/membrane/envelope biogenesis |
| N840_1771 | chbR | 2.006 | ENOG410XRRP | K:Transcription |
| N840_1491 | yddH | 2.039 | COG1853 | S:Function unknown |
| N840_0603 | fepC | 4.56 | COG1120 | M:Cell wall/membrane/envelope biogenesis |
| N840_4364 | fecE | 2.583 | COG1120 | L:Replication, recombination and repair |
| N840_3320 | yhdV | 7.212 | ENOG41124RN | M:Cell wall/membrane/envelope biogenesis |
| N840_0747 | ybgT | 2.017 | COG4890 | S:Function unknown |
| N840_0706 | kdpE | 2.054 | COG0745 | T:Signal transduction mechanisms |
| N840_3605 | yhjX | 2.416 | ENOG410XQUK | G:Carbohydrate transport and metabolism |
| N840_1024 | rutA | 2.962 | COG2141 | J:Translation, ribosomal structure and biogenesis |
| N840_0709 | kdpB | 2.102 | COG2216 | P:Inorganic ion transport and metabolism |
| N840_3542 | yhiJ | 2.371 | ------ | ------ |
| N840_1399 | ynaE | 3.158 | ENOG411246H | K:Transcription |
| N840_3626 | xylB | 2.074 | COG1070 | S:Function unknown |
| N840_0148 | fhuC | 2.201 | COG1120 | P:Inorganic ion transport and metabolism |
| N840_0341 | prpB | 3.579 | COG2513 | G:Carbohydrate transport and metabolism |
| N840_0551 | ybcL | 2.042 | COG1881 | S:Function unknown |
| N840_1053 | ymdA | 4.16 | ENOG410Y0RF | S:Function unknown |
| N840_1028 | efeU | 2.206 | COG0672 | P:Inorganic ion transport and metabolism |
| N840_0599 | fes | 4.516 | COG2382 | P:Inorganic ion transport and metabolism |
| N840_3168 | tdcB | 2.845 | COG1171 | S:Function unknown |
| N840_2074 | insH1 | 3108.029 | COG3039 | L:Replication, recombination and repair |
| N840_2892 | omrA | 3.084 | ------ | ------ |
| N840_1052 | csgC | 31.665 | ENOG4111VMB | O:Posttranslational modification, protein turnover, chaperones |
| N840_1989 | rcsA | 2.784 | COG2771 | N:Cell motility |
| N840_1381 | ydaG | 2.282 | ------ | ------ |
| N840_0342 | prpC | 3.307 | COG0372 | C:Energy production and conversion |
| N840_1509 | bdm | 6.597 | ENOG4111V9Q | S:Function unknown |
| N840_1034 | pgaB | 2.207 | COG0726 | M:Cell wall/membrane/envelope biogenesis |
| N840_1995 | yodC | 3.063 | COG5475 | S:Function unknown |
| N840_2504 | eutH | 2.473 | COG3192 | E:Amino acid transport and metabolism |
| N840_4192 | adiY | 3.69 | ENOG4110B0F | K:Transcription |
| N840_3211 | mtr | 2.439 | COG0814 | I:Lipid transport and metabolism |
| N840_4074 | zraP | 4.796 | COG3678 | S:Function unknown |
| N840_1119 | ycfJ | 2.409 | COG3134 | M:Cell wall/membrane/envelope biogenesis |
| N840_0718 | ybfL | 2.886 | COG5433 | L:Replication, recombination and repair |
| N840_0604 | fepG | 3.936 | COG4779 | P:Inorganic ion transport and metabolism |
| N840_4412 | yjiG | 2.13 | COG0700 | S:Function unknown |
| N840_4366 | fecC | 2.058 | COG0609 | L:Replication, recombination and repair |
| N840_2801 | ygbE | 2.208 | ENOG4111VSF | S:Function unknown |
| N840_0716 | ybfC | 2.991 | ENOG410YGXJ | S:Function unknown |
| N840_0273 | yagA | 4.424 | ENOG410XTHD | L:Replication, recombination and repair |
| N840_4398 | fimI | 2.311 | COG3539 | S:Function unknown |
| N840_3556 | arsB | 2.154 | COG1055 | P:Inorganic ion transport and metabolism |
| N840_0732 | ybgD | 2.805 | ENOG410YS65 | G:Carbohydrate transport and metabolism |
| N840_2756 | srlB | 4.334 | COG3731 | G:Carbohydrate transport and metabolism |
| N840_4369 | fecR | 2.46 | COG3712 | T:Signal transduction mechanisms |
| N840_0075 | leuL | 2.064 | ------ | ------ |
| N840_0343 | prpD | 2.321 | COG2079 | S:Function unknown |
| N840_3510 | livH | 2.76 | COG0559 | E:Amino acid transport and metabolism |
| N840_1544 | lsrF | 3.307 | COG1830 | G:Carbohydrate transport and metabolism |
| N840_2041 | yeeS | 2.972 | COG2003 | S:Function unknown |
| N840_1421 | paaG | 2.04 | COG1024 | S:Function unknown |
| N840_0714 | ybfB | 2.852 | ENOG410ZEKG | S:Function unknown |
| N840_3558 | yhiS | 8.7 | COG3039 | L:Replication, recombination and repair |
| N840_1543 | lsrB | 3.199 | ENOG410XPHQ | G:Carbohydrate transport and metabolism |
| N840_1608 | ydfE | 3.09 | ENOG4111NBJ | S:Function unknown |
| N840_3380 | gspF | 3.194 | COG1459 | U:Intracellular trafficking, secretion, and vesicular transport |
| N840_0597 | entD | 2.244 | COG2977 | Q:Secondary metabolites biosynthesis, transport and catabolism |
| N840_4296 | ytfI | 2.038 | ------ | ------ |


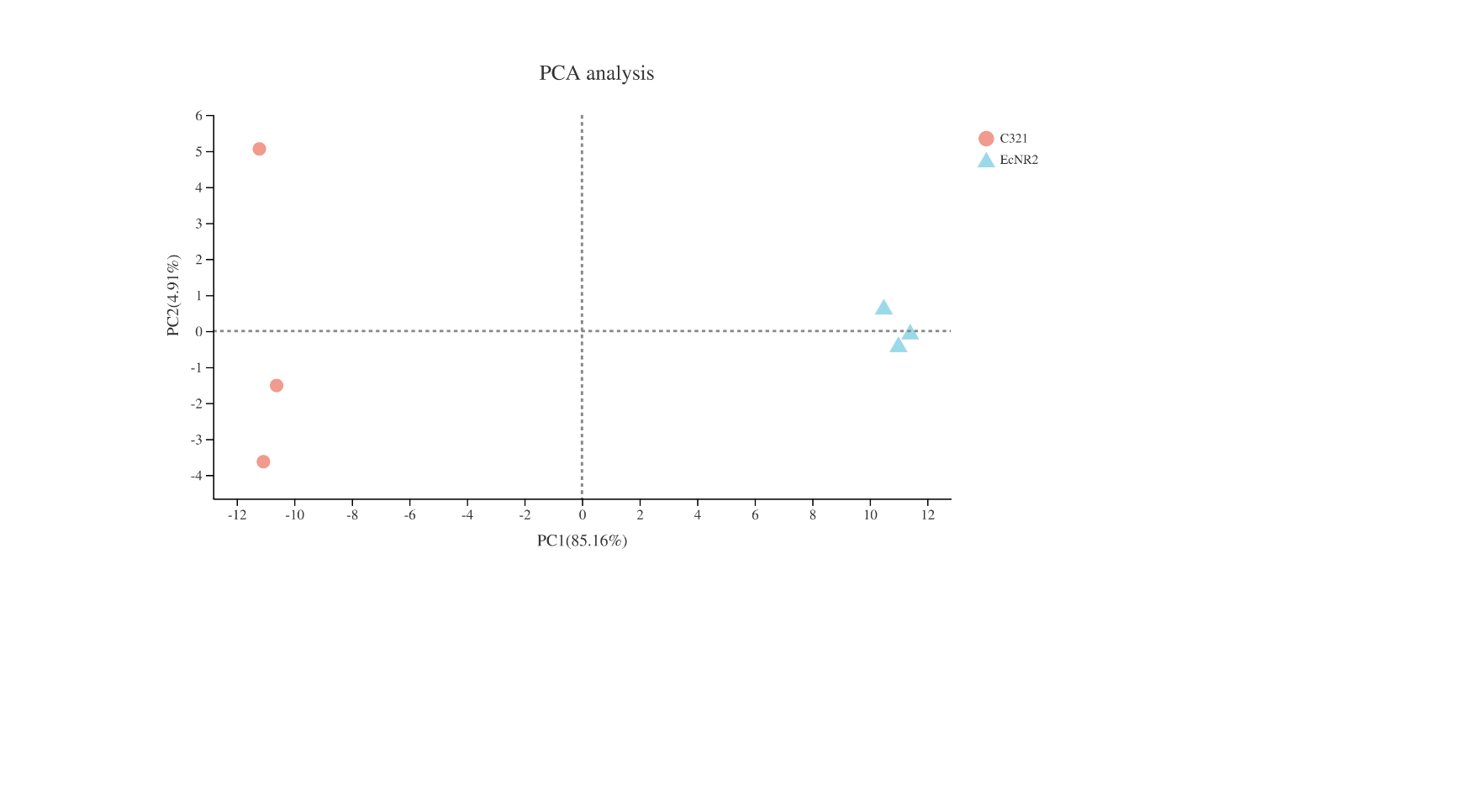


**Figure S1. Principal co-ordinates analysis (PCoA) of the two strain samples. Principal component 1 (PC1) and 2 (PC2) explained 85.16% and 4.91% of the variance, respectively. Distances between each symbol in the plot reflect relative dissimilarities of transcriptome of each samples.**


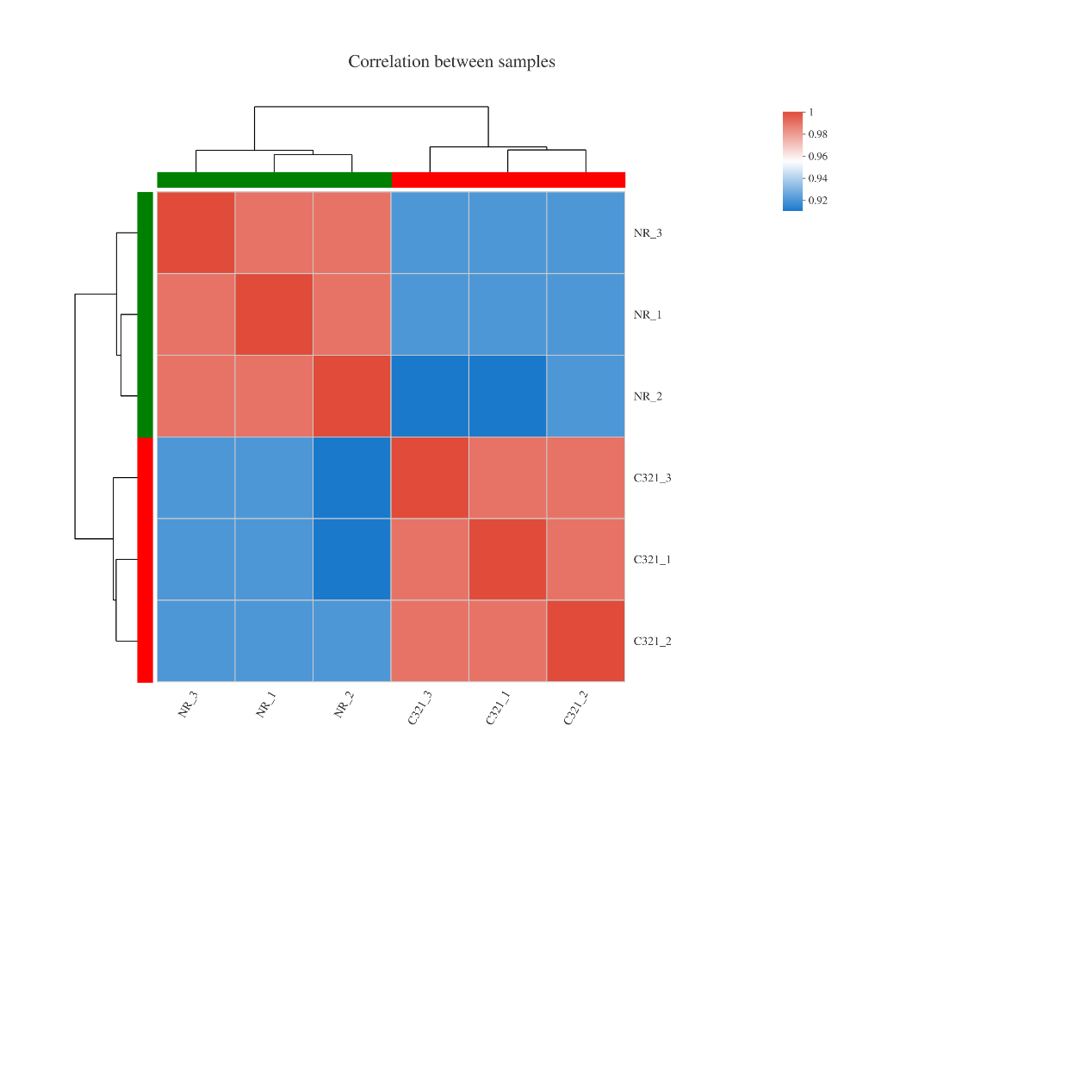


**Figure S2. Correlation coefficient analysis of the relationship of between each sample**


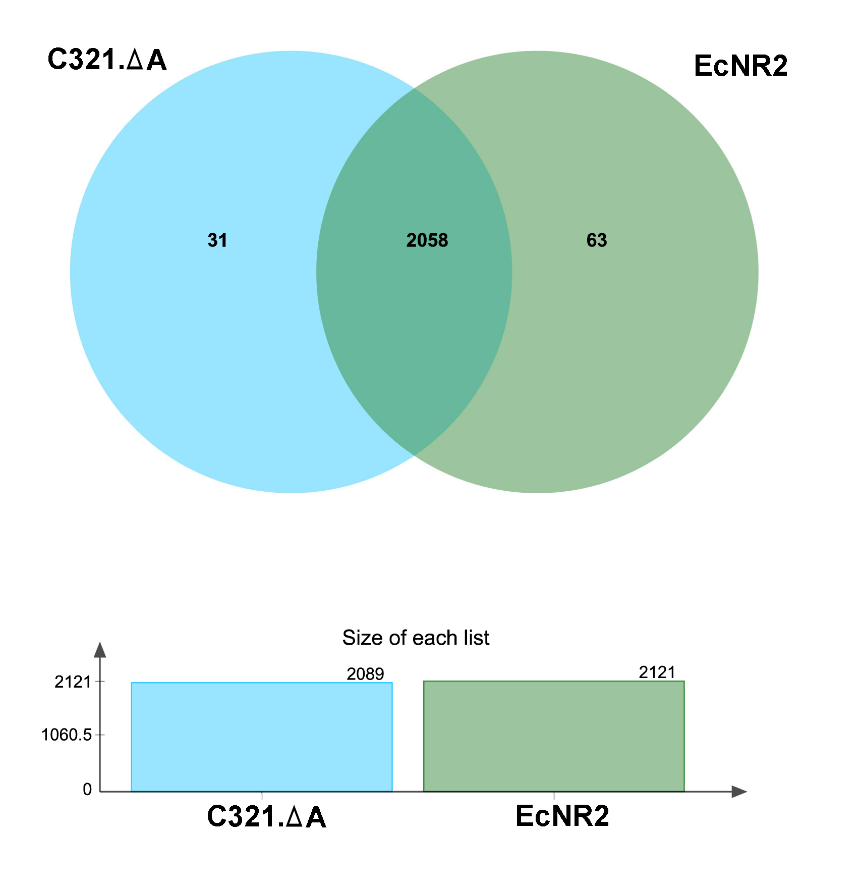


**Figure S3. A Venn diagram of shared and unique of different expressed proteins between *E.coli* C321.ΔA and its parent strain.**


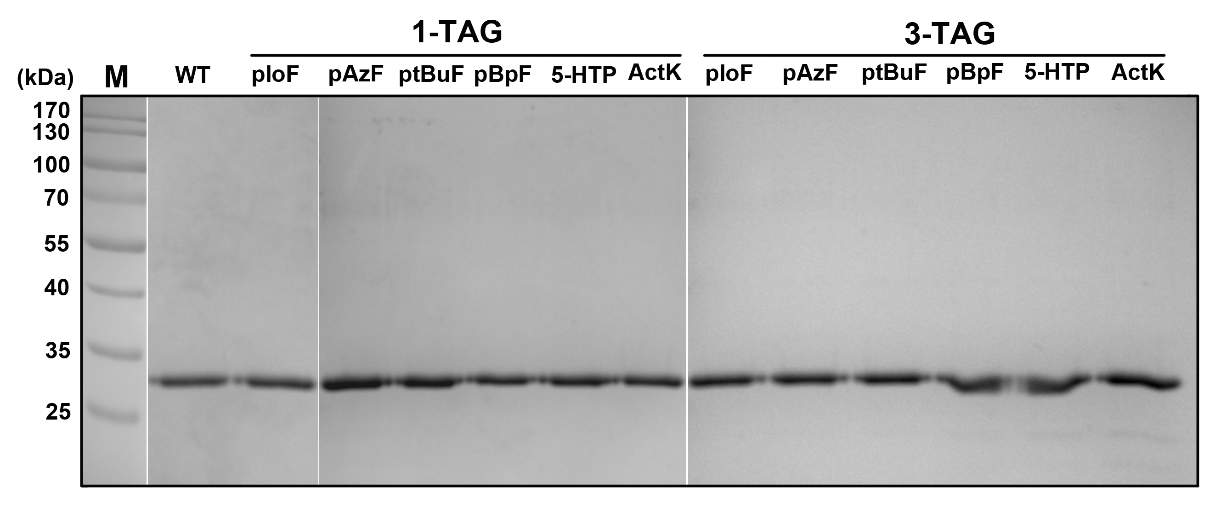


**Figure S4. SDS-PAGE analysis of purified samples of the GFP variants.**
